# Conjugated Microporous Polymers for Catalytic CO_2_ Conversion

**DOI:** 10.1002/advs.202308228

**Published:** 2024-02-07

**Authors:** Ulzhalgas Karatayeva, Safa Ali Al Siyabi, Basiram Brahma Narzary, Benjamin C. Baker, Charl F. J. Faul

**Affiliations:** ^1^ School of Chemistry University of Bristol Bristol BS8 1TS UK

**Keywords:** chemical conversion, CO_2_ reduction, conjugated microporous polymers, cyclic carbonates, cycloaddition reaction, electrocatalysts, epoxides

## Abstract

Rising carbon dioxide (CO_2_) levels in the atmosphere are recognized as a threat to atmospheric stability and life. Although this greenhouse gas is being produced on a large scale, there are solutions to reduction and indeed utilization of the gas. Many of these solutions involve costly or unstable technologies, such as air‐sensitive metal–organic frameworks (MOFs) for CO_2_ capture or “non‐green” systems such as amine scrubbing. Conjugated microporous polymers (CMPs) represent a simpler, cheaper, and greener solution to CO_2_ capture and utilization. They are often easy to synthesize at scale (a one pot reaction in many cases), chemically and thermally stable (especially in comparison with their MOF and covalent organic framework (COF) counterparts, owing to their amorphous nature), and, as a result, cheap to manufacture. Furthermore, their large surface areas, tunable porous frameworks and chemical structures mean they are reported as highly efficient CO_2_ capture motifs. In addition, they provide a dual pathway to utilize captured CO_2_ via chemical conversion or electrochemical reduction into industrially valuable products. Recent studies show that all these attractive properties can be realized in metal‐free CMPs, presenting a truly green option. The promising results in these two fields of CMP applications are reviewed and explored here.

## Graphical abstract

Conjugated microporous polymers (CMPs) enable the chemical conversion or electrochemical reduction of CO_2_, resulting in the production of industrially valuable products. Recent studies demonstrate that these desirable properties can be attained in metal‐free CMPs, offering a route to sustainable and green long‐term solutions. This review provides an overview of the use of these promising materials to address globally significant challenges.

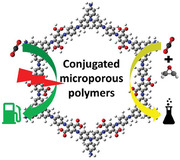

## 1 Introduction

### 1.1 Impact of CO_2_ on Climate Change

The reliance on fossil fuel resources, dating back to the Industrial Revolution, has led to drastically increasing energy demands, and a resulting increase in anthropogenic greenhouse gas emissions, particularly carbon dioxide (CO_2_), in the atmosphere.^[^ ^1^, ^2^ ^]^ At present, the National Oceanic and Atmospheric Administration (NOAA) has measured the average concentration of CO_2_ at 419 parts per million (ppm) in October 2023, far surpassing the pre‐industrial levels.^[^ ^3^ ^]^ Scientists within the Intergovernmental Panel on Climate Change (IPCC), through extensive statistical analyses, have asserted that this rise in CO_2_ levels is primarily attributable to human activity, particularly fossil fuel combustion, and is leading to severe climate changes.^[^ ^4^ ^]^ The IPCC projected a probable rise in CO_2_ levels to ≈953 ppm by 2100, and this scenario would result in a range of temperature increases from 2.6 to 4.8 °C above pre‐industrial levels.^[^ ^5^ ^]^ Regrettably, worldwide CO_2_ emissions arising from fossil fuel burning activities are expected to soar to 45.5 billion tons per annum by 2040.^[^ ^6^ ^]^ Nevertheless, despite the detrimental impacts of fossil fuel consumption on the environment and human health, the slow growth of appropriate technological options for renewable energy has led to the continued usage of fossil fuel resources worldwide as primary energy source.^[^ ^7^ ^]^

### 1.2 Importance of CO_2_ Capture and Conversion

On a more positive note, CO_2_ is crucial for growth of plants (as very effective carbon capture mechanism) and has wide‐ranging applications in many industrial processes.^[^ ^8^ ^]^ For instance, CO_2_ is a relatively inexpensive, abundant, nontoxic, and renewable C1 building block with significant potential in the synthesis of a multitude of high‐value chemicals, feedstocks, and fuels, including organic carbonates,^[^ ^9^, ^10^ ^]^ formamides,^[^ ^11^ ^]^ carboxylic acids,^[^ ^12^ ^]^ alkyl amines,^[^ ^13^ ^]^ urea derivatives,^[^ ^14^, ^15^ ^]^ and alcohols.^[^ ^16^, ^17^ ^]^

CO_2_ capture marks the initial phase in removing CO_2_ from the atmosphere, with technologies for capture being commercially available since the 1950s.^[^ ^18^, ^19^ ^]^ The main challenge today is the fate of the captured CO_2_. Storing it deep underground or dissolving it in the mid‐depth oceanic waters are some of the proposed solutions for long‐term storage.^[^ ^19^ ^]^ However, concerns regarding leakage, high energy processes to realize these solutions, and consequent unsustainability have caused some reluctance in adopting such CO_2_ capture and storage strategies throughout the world.^[^ ^19^ ^]^

The previous emphasis on CO_2_ capture and storage (CCS) to decrease atmospheric CO_2_ levels has shifted toward CO_2_ capture and utilization (CCU), as it is deemed more efficient and environmentally friendly.^[^ ^20^ ^]^ The optimal approach to reduce global warming while also tackling the energy crisis is to combine these two technologies, known as CO_2_ capture, utilization, and storage (CCUS) solutions, which will be discussed in detail later in this review.^[^ ^21^ ^]^

To achieve sustainable development, it would be critical for researchers to focus on developing advanced materials with two key properties. First, the materials should be able to absorb CO_2_ effectively. Second, such materials should also be able to act as catalysts for the conversion of CO_2_ into fuels. Developing these properties pose significant challenges for the scientific community to ensure a sustainable future. Although there have been many exciting developments in the field of CCUS materials and their use in reducing CO_2_ emissions, achieving large‐scale conversion of CO_2_ continues to pose a significant challenge for the future.^[^ ^22^ ^]^ Despite the progress made in this area, there are still many technical and financial hurdles that must be overcome to make these technologies economically viable and practical for widespread use. As a result, more research and investment is needed in this field before it can reach its full potential.

**Scheme 1:**
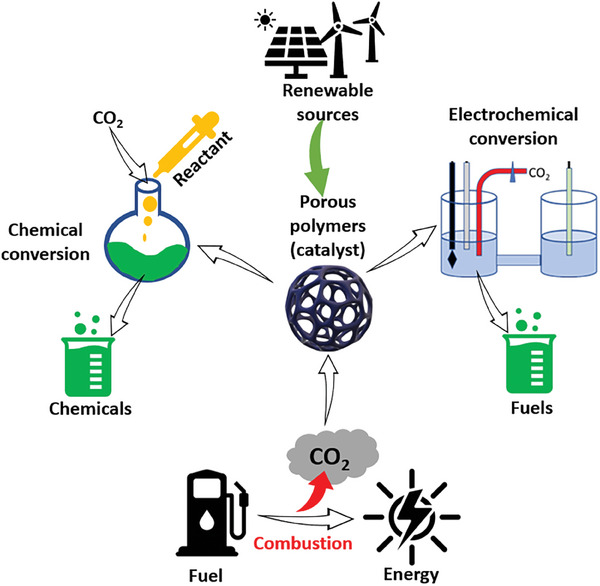
Carbon capture and routes to CO_2_ conversion using porous materials.

There are several ways to convert CO_2_ into useful materials, including reforming,^[^ ^23^, ^24^ ^]^ biotransformation,^[^ ^25^, ^26^ ^]^ chemical methods,^[^ ^27^ ^]^ thermochemical catalysis,^[^ ^28^ ^]^ photothermal conversion,^[^ ^29^ ^]^ photocatalytic^[^ ^30^, ^31^, ^32^, ^33^ ^]^ and electrocatalytic reduction.^[^ ^34^, ^35^ ^]^ However, in this review we specifically focus on recently published chemical and electrochemical conversion studies, **Scheme** **1**, with a broader focus on studies published since 2013. Other conversion routes are not discussed in this review. The authors refer readers to other reviews and studies that address these topics in detail.^[^ ^23^, ^24^, ^25^, ^26^, ^28^, ^29^, ^30^ ^]^

### 1.3 CMPs as Potential CO_2_ Conversion Catalysts

There has been growing interest in conjugated microporous polymers (CMPs), which are materials with attractive properties including tunability and expanded π‐conjugation.^[^ ^36^, ^37^, ^38^, ^39^, ^40^ ^]^

CMPs are proving to be a versatile and powerful tool for addressing a wide range of environmental and energy related challenges. As a result, they are used in a variety of practical applications, including as adsorbents,^[^ ^41^, ^42^, ^43^ ^]^ heterogeneous catalysts,^[^ ^9^, ^44^, ^45^ ^]^ energy storage,^[^ ^46^, ^47^ ^]^ luminescent materials^[^ ^48^ ^]^ and light harvesting.^[^ ^49^ ^]^

CMPs offer numerous advantages over other porous materials such as zeolites, covalent organic frameworks (COFs), metal‐organic frameworks (MOFs), and activated carbons. One primary advantage of CMPs is the versatility of available synthesis approaches.^[^ ^50^, ^51^ ^]^ While COFs are typically produced through high‐temperature condensation reactions at small scale, CMPs can be synthesized using various metal‐catalyzed couplings (including Sonogashira‐Hagihara, Suzuki, and Buchwald‐Hartwig) to metal‐free condensation reactions at much larger scale.^[^ ^51^, ^52^ ^]^ In addition, the majority of reactions employed in COF synthesis exhibit reversibility at high temperatures, thereby restricting the selection of functional building units to those capable of withstanding these extreme conditions.^[^ ^53^, ^54^ ^]^

However, the key distinction between COFs and CMPs lies in their structural nature: COFs are crystalline and CMPs are amorphous, giving CMPs greater design flexibility. This property of CMPs opens up more design possibilities, allowing for the creation of multi‐component CMP catalysts through multi‐step tandem reactions, which are challenging to achieve with COF materials.^[^ ^36^, ^37^ ^]^ Moreover, highly ordered materials like COFs often form multiple interpenetrated networks, which can pose a challenge and lead to obstruction of the available pores.^[^ ^53^ ^]^ From a molecular design perspective, one of the distinguishing features of CMPs is the broad diversity of building blocks that can be employed.^[^ ^55^ ^]^ This modular framework construction allows for well‐accessible pores, sometimes even enabling the creation of hierarchical pore systems to enhance transport kinetics.^[^ ^53^ ^]^

Furthermore, significant advances have recently been made in the use of CMPs for heterogeneous catalysis.^[^ ^55^ ^]^ Utilizing a bottom‐up approach, catalytic moieties can be directly incorporated into the CMP framework to produce heterogeneous CMP catalysts. Similar to COFs, the resulting materials feature high surface areas and a uniform distribution of catalytic sites, offering the potential for high catalytic activity.^[^ ^55^ ^]^

In contrast to well‐established catalysts and support materials such as MOFs,^[^ ^56^, ^57^ ^]^ and activated carbons (ACs),^[^ ^58^, ^59^ ^]^ CMPs present a promising avenue for advancing catalytic processes in the future. One notable distinction between CMPs and MOFs is the superior chemical stability of CMPs, which is particularly crucial for catalytic applications. CMPs combine the modular construction features of MOFs with the robust stability of polymers, thanks to the presence of covalent bonds. Activated carbons, on the other hand, typically offer a heterogeneous surface that can only be moderately adjusted through variations in the activation process or post‐synthetic chemical treatments. CMPs, with their modular building block approach, offer highly tunable surface chemistries through organic chemistry methods, presenting a potential solution to address most of the aforementioned limitations.^[^ ^53^ ^]^

CMPs are unique in their ability to combine various desirable properties, making them a compelling addition to conventional porous materials.^[^ ^51^ ^]^ They encompass the characteristics of both modular homogeneous and recyclable heterogeneous catalysts, offering a versatile, robust and efficient platform for catalytic applications.^[^ ^53^, ^54^ ^]^ This versatility positions CMPs as contributors to a greener and more sustainable future by enabling the development of more efficient and eco‐friendly processes.^[^ ^53^ ^]^

Drawing from these underlying principles, the targeted design and synthesis of CMPs with one or multiple types of active sites to facilitate the capture and catalytic conversion of CO_2_ is well within reach.

For a more comprehensive understanding of why CMPs are increasingly considered the preferred catalyst, the reader is referred to detailed review papers^[^ ^37^, ^53^, ^54^, ^55^ ^]^ that provide in‐depth insights into the structural attributes and advantages of CMPs as catalysts. Additionally, readers are referred to recent excellent and comprehensive reviews,^[^ ^34^, ^60^, ^61^, ^62^ ^]^ where other POPs were applied for catalytic CO_2_ conversion.

## 2 Chemical Conversion

**Scheme 2:**
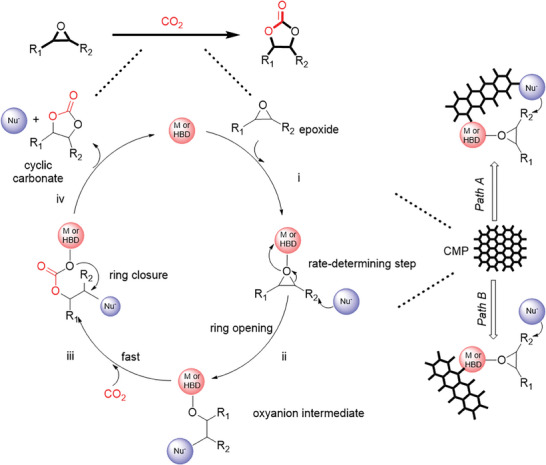
The general epoxide activation catalytic mechanism for the formation of cyclic carbonates via cycloaddition reaction of CO_2_ and epoxides. (Red: M – metal center or HBD – hydrogen‐bond donor group; Blue: Nu^−^ – nucleophilic group).

The chemical conversion of CO_2_ is particularly appealing to many researchers to not only mitigate global warming, but to explore green and sustainable routes toward a wide variety of commodity polymers, chemicals, and feedstocks for further chemical processes. Non‐redox pathways to convert CO_2_ may be utilized as a potential approach given its 100% atom‐economy, sustainable, and environmentally friendly manufacturing. In general, chemical reactions that incorporate CO_2_ as a building block can often be carried out under mild conditions (**Scheme** **2**).^[^ ^4^, ^63^ ^]^ These properties present CMPs as an attractive avenue for sustainable and environmentally friendly chemistry. For example, when CO_2_ is combined with epoxides, the resulting cyclic‐ and polycarbonates can be very useful and much safer to produce than those made with phosgene.^[^ ^64^ ^]^ Urea and other compounds that are commonly used in fertilizers can also be synthesized directly from CO_2_ and ammonia/amines, which is another advantage of this method.^[^ ^14^ ^]^

### 2.1 Fundamental Principles of the Chemical Conversion of CO_2_

The cycloaddition reaction of CO_2_ with epoxides to produce cyclic carbonates (widely used in the production of pharmaceuticals and fine chemicals, electrolytes for lithium‐ion batteries, raw materials for plastics, and as nontoxic solvents) is regarded as one of the most alluring processes for CO_2_ fixation.^[^ ^65^ ^]^ Hence, numerous industrial processes have been developed for the manufacturing of carbonates via cycloaddition of CO_2_ with epoxides.^[^ ^64^ ^]^

In general, there are three main categories of mechanisms for synthesizing cyclic carbonates: epoxide activation, CO_2_ activation, and dual activation involving both epoxide and CO_2_.^[^ ^66^ ^]^ Epoxide activation is the most commonly proposed mechanism, which involves a series of steps outlined in Scheme 2. The endothermic cycloaddition of CO_2_ with an epoxide in presence of catalyst involves four stages to form cyclic carbonates, which follows pseudo first‐order kinetics. The four stages are as follows (shown in Scheme 2): i) coordination of the oxygen atom of the epoxide either with the electropositive metal (e.g., Zn, Co, Cu etc., in the case of metal‐based catalysts) or a hydrogen‐bond donor (HBD) group (e.g., amines, carboxylic acids etc., in the case of metal‐free catalysts), ii) ring opening via a nucleophilic attack, originating from either the co‐catalyst or the catalyst‐containing nucleophilic sites, on the least hindered carbon atom of the activated epoxide. This step results in the formation of an oxyanion intermediate, which is regarded as the most challenging step due to the high activation energy.^[^ ^9^ ^]^ The role of the nucleophile is to initiate the opening of the three‐membered ring. The third stage iii) involves the rapid insertion of CO_2_, producing an alkyl carbonate anion. And the final stage iv) is the formation of the cyclic carbonate by an intramolecular ring closure. The nucleophile leaves the product with simultaneous desorption of product from the catalyst, leading to the termination of the catalytic cycle and the regeneration of the catalyst (Scheme 2).^[^ ^67^ ^]^

**Scheme 3:**
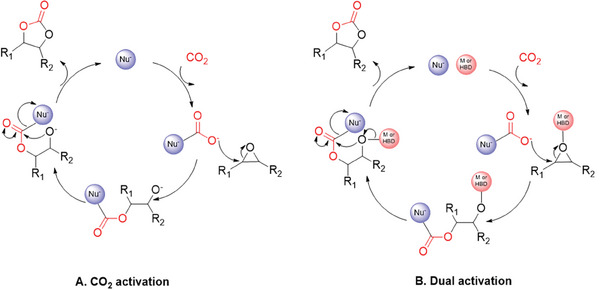
Other possible catalytic mechanism pathways for the formation of cyclic carbonates via cycloaddition reaction of CO_2_ and epoxides through A. CO_2_ activation and B. Dual activation (where both Nu^−^ and M or HBD are attached to CMP as shown in Scheme 2).

In this review, all reported catalytic systems except for Sharma et al. propose the pathway through epoxide activation (Scheme 2). Sharma et al.^[^ ^68^ ^]^ suggest an alternative reaction mechanism involving CO_2_ activation, where the nucleophile attacks the electrophilic carbon on CO_2_ (**Scheme** **3**A). In addition, in 2021, Guo et al.^[^ ^66^ ^]^ published a review discussing the development of catalysts capable of simultaneously activating both carbon dioxide and epoxide (Scheme 3B). They emphasize that the advantage of the dual activation mechanism lies not only on achieving higher rate enhancements but also in controlling the stereochemistry of the cyclic carbonate product.

**Figure 1:**
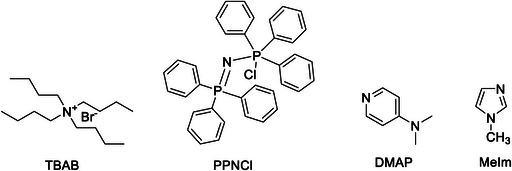
Structures of common co‐catalysts used in cycloaddition of CO_2_ to epoxides.

As mentioned, the ring‐opening step, Scheme 2, Step ii, is the key step.^[^ ^69^ ^]^ This rate‐determining step of the cycloaddition reaction between epoxides and CO_2_ is the target of many catalysts. Prior to the year 2000, tetrabutylammonium bromide (TBAB, **Figure** **1**) was commonly employed as a homogeneous catalyst in industrial applications.^[^ ^67^ ^]^ However, the harsh reaction conditions required for the cycloaddition of CO_2_ (T>220°C, P_CO2_ >8.0 MPa), as well as the instability and toxicity of the catalyst, resulted in a complex technological process and low‐quality colored products. Furthermore, complex separation steps are often necessary for the separation of the homogenous catalyst from the formed products.

Heterogeneous catalysts present a method to bypass these separation issues; however, they often exhibit poor catalytic activity for this process.^[^ ^27^ ^]^ The heterogeneous catalysts that have been reported to date have only demonstrated effectiveness for terminal epoxides, making it a significant challenge to develop a catalyst capable of converting internal epoxides. Moreover, despite the simplicity of purification and recyclability offered by heterogeneous catalysts, most catalysts require high temperatures and extreme CO_2_ pressures to function effectively.^[^ ^70^, ^71^ ^]^ Only a small number of catalysts have the capacity to convert CO_2_ into cyclic carbonates under relatively benign conditions, as shown, for example, in recent publications by Zhang et al.,^[^ ^10^ ^]^ Ding et al.^[^ ^72^ ^]^ and Narzary et al.^[^ ^73^ ^]^

To create multi‐functional heterogeneous catalysts, for which significant need exists,^[^ ^27^ ^]^ the following factors should be taken into account:

1) selecting a simple, sustainable, and high‐yield synthetic approach to synthesize the catalyst.
2) incorporating electrophilic and nucleophilic groups capable of activating both epoxides and CO_2_.
3) incorporating different CO_2_‐philic moieties into catalyst, such as heteroatoms and ionic groups, to enhance CO_2_ uptake ability and guarantee CO_2_ enrichment close to active sites.
4) exhibiting a high specific surface area and hierarchical porous structures, i.e., possessing a sufficient number of micropores to improve CO_2_ adsorption and a large number of meso‐ and macropores to promote efficient diffusion and mass transport of substrates and products.
5) changing the electronic and steric characteristics of active centers and/or ligands to improve the efficiency of catalysts.

Porous materials, and in particular CMPs, present a simple method to design heterogeneous catalysts that incorporate these 5 points. It is worth noting that many systems often require the use of homogeneous additives as co‐catalysts (Figure 1) to facilitate ring opening during cyclization and enhance overall catalytic efficiency.

### 2.2 CMPs for the Chemical Conversion of CO_2_

CMP‐based catalysts can either be metal‐containing or metal‐free (see Pathways A and B in Scheme 2). The ability to transform CO_2_ into cyclic carbonates by CMP‐based catalysts can be divided into two groups:

1) Binary catalytic systems with a nucleophilic co‐catalyst (dispersed through the network but not attached) and CMPs (Scheme 2, Path A).

Binary catalytic systems have a disadvantage due to the necessity of using homogeneous co‐catalysts, which results in only partial heterogeneity of the catalyst; it is necessary to add extra additives (e.g., fresh TBAB) to the reaction mixture during the catalyst recycling process. The complexity and higher expense of catalyst recovery and product purification make this approach less attractive.^[^ ^27^ ^]^

2) one‐component catalytic systems, which comprise of CMPs that possess nucleophilic sites covalently attached to the network (Scheme 2, Path B).

One‐component bifunctional systems can make the catalyst recovery processes easier and boost catalytic efficiency. The incorporation of nucleophiles has been recognized as a highly effective approach to heterogeneous CO_2_ catalysis since they do not necessitate the use of (additional) homogeneous co‐catalysts, thereby rendering the process genuinely heterogeneous (without the need to reload co‐catalyst).^[^ ^27^ ^]^

We first discuss the conversion of propylene oxide (PO) to propylene carbonate (PC) using both metal‐based and non‐metal‐based binary and one‐component systems, as shown in **Table** **1**. In the next section (Section 2.2.3) the conversion of other epoxides (see Figure 5) into their corresponding cyclic carbonates is discussed.

**Table 1:** The catalytic performance for the chemical conversion of CO_2_ catalyzed by various catalytic systems using propylene oxide (PO) as a model substrate.

Entry	Polymeric material	BET [m^2^g^−1^]	CO_2_ uptake [wt.%]	*t* [h]	*T* [°C]	*P* CO_2_ [MPa]	Yield [%]	no. of runs	TOF [h^−1^]	TON	References
1	Co‐CMP^a)^	965	7.93^c)^	48 1	25 100	0.1 3	82 98	22 1	3.5 201	167 201	[9]
2	Al‐CMP^a)^	798	7.65^c)^	48 1	25 100	0.1 3	78 91	3 1	3.3 187	160 187	
3	Zn‐CMP^a)^	791	5.84^c)^	48 1	25 120	0.1 3	76 94	10 1	8.0 470	384 470	[64]
4	Cr‐CMP^a)^	738	7.17^c)^	48 1	25 100	0.1 3	68 99	10 1	3.2 224	154 224	[74]
5	Zn@SBMMP^a)^	423	9.40^d)^	4	80	2	95	1	50.8	203	[75]
6	Al‐CMP^b)^	839	2.74^c)^ 4.30^d)^	5 5	70 100	3 3	74 91	5 1	296.0 364	1480 1820	[76]
7	PCP‐Cl	755	6.14^c)^ 10.17^d)^	12	100	3	99	4	n.r.	n.r.	[77]
8	HUST‐1‐Co^a)^	1360	13.17^c)^ 21.39^d)^	30	25	0.1	95	15	103.0	3101	[78]
9	Co@PDVB‐VP‐0.5^a)^	479	6.51^c)^ 8.10^d)^	48 0.75	30 100	1^e)^ 1^e)^	99 99	5 1	16.2 1034.3	778 776	[79]
10	Co‐CMP‐2^a)^	475	6.46^c)^	1	100	3	99	10	236.0	236	[80]
11	Al‐iPOP‐1	52	5.80^d)^	3	40	1	97	6	892.0	2676	[81]
12	Al‐iPOP‐`2	86	6.60^d)^				99	6	916.0	2748	
13	POF‐PNA‐Br^−^	288	n.r.	48 8	40 60	0.1 1	98 >99	3 1	7.0 42.6	337 341	[82]
14	IPF‐CSU‐1^a)^	n.r.	n.r.	48	25	0.1	99	3	n.r.	n.r.	[83]
15	p‐TBIB^a)^	840	12.31^c)^ 19.79^d)^	24	25	0.1	97	10	9.9	237	[72]
16	CPBr‐2	370	8.81^d)^	7	90	2.5	95	5	n.r.	n.r.	[84]
17	SCHPP‐3^a)^	518	5.77^d)^	5	120	2	93	1	n.r.	n.r.	[71]
18	DTBBQ‐CMP^a)^	16	n.r.	48	25	0.1	99	5	60.5	2903	[85]
19	Zn‐salen‐CMP^a)^	589	4.15^c)^ 6.58^d)^	1.5	120	3	92	8	307.0	461	[70]
20	HMP‐TAPA^a)^	424	10.67^d)^	6	80	0.6	>99	1	78.7	472	[68]
21	TBB‐BPY‐Co^a)^	1279	8.19^c)^ 15.73^d)^	48	25	1	85	10	306.0	14 680	[86]
22	CMP‐Salen‐Zn^a)^	444.6	4.04^c)^ 5.95^d)^	12	120	0.1	99	1	n.r.	n.r.	[87]
23	Co‐Por‐POP‐2^a)^	780	9.83^c)^ 15.10^d)^	48 48	25 25	0.1 1	73 89	10 1	20.8 25.6	997 1227	[88]
24	Al‐Por‐POP‐2^a)^	1037	10.31^c)^ 17.12^d)^	48 1	25 100	0.1 3	95 99	10 1	65.5 3261.0	3142 3261	[10]
25	HVPOP‐Br	266.7	≈4.1^c)^ ≈6.5^d)^	12	120	1	93	1	n.r.	n.r.	[89]

^a)^ TBAB co‐catalyst;

^b)^ PPNCl co‐catalyst (see Figure 1);

^c)^ 298 K;

^d)^ 273 K; n.r. = not reported; TOF = Turnover frequency; TON = Turnover number; t = reaction time; T = reaction temperature; P = reaction pressure;

^e)^ CO_2_:N_2_ at 0.15:0.85.

#### 2.2.1 Metal‐Based CO_2_ Cycloaddition Reactions

**Figure 2:**
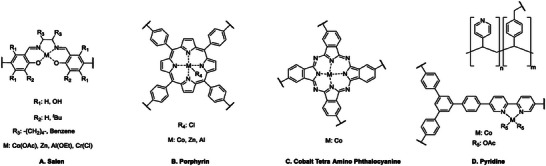
Common structures of metal binding sites within CMPs exploited for the catalyzed cycloaddition of CO_2_ to epoxides.

Owing to their ability to coordinate with the O atom of epoxides (Scheme 2‐i), Lewis acid metal sites such as Zn^2+^, Co^2+^, Al^3+^ have demonstrated good performance in catalyzing the cycloaddition of CO_2_ to epoxides. Numerous metalated CMPs with coordination units such as porphyrin,^[^ ^76^ ^]^ salen,^[^ ^9^ ^]^ pyridine^[^ ^82^ ^]^ (**Figure** **2**) have therefore been designed over the years to exploit this approach.

##### Salen‐Based CMPs

Salens, as shown in Figure 2, are known to exhibit a high affinity for binding metal ions,^[^ ^62^ ^]^ making them efficient in homogeneous systems.^[^ ^6^, ^90^ ^]^ In heterogeneous systems, binary catalysts of CMPs with salen ligands and coordinated metals have been used to effectively transform CO_2_ into cyclic carbonates by reacting with epoxides in the presence of a TBAB co‐catalyst (Scheme 2, Path A).^[^ ^9^, ^64^, ^74^ ^]^

In 2013, Deng et al. were the first to show a range of metal‐functionalized CMPs (containing Cr, Zn, Al, and Co) that were synthesized through a Sonogashira‐Hagihara (SH) cross‐coupling reaction between dibromo metallosalen complexes and 1,3,5‐triethynylbenzene to yield Compound A, Figure 2.^[^ ^9^ ^]^ The reported Cr‐salen‐containing CMP (Cr‐CMP) is capable of capturing CO_2_ (7.17 wt.%), which is typically a prerequisite for the storage and utilization of CO_2_.^[^ ^74^ ^]^ The Cr‐CMP exhibits exceptional catalytic activity of 99% for the cycloaddition reaction of CO_2_ with PO (> 96.1% conversion for different terminal epoxides, see Section 2.2.3) resulting in the formation of cyclic carbonates at 100 °C and 3.0 MPa of CO_2_. The catalyst was reused more than ten cycles without changes in its catalytic activity.

The same research group developed a cobalt‐coordinated CMP known as Co‐CMP, which demonstrated a substantial BET specific surface area (965 m^2^ g^−1^) and a reasonable CO_2_ uptake (7.93 wt.%).^[^ ^9^ ^]^ Its adsorption capacity was comparable to that of certain previously reported inorganic catalysts^[^ ^91^ ^]^ and MOFs.^[^ ^92^ ^]^ The authors conducted a comparison between the catalytic performance of Co‐CMP and industrial catalysts like potassium iodide (KI) and potassium iodide/β‐cyclodextrin (KI/β‐CD) that are used for PO/CO_2_ coupling.^[^ ^93^ ^]^ Under atmospheric pressure and room temperature, KI and KI/β‐CD exhibited poor catalytic activities with yields of 3.8% and 3.9% respectively. In contrast, Co‐CMP achieved a yield of 82% under the same conditions (Table 1, Entry 1). To further demonstrate the superiority of the heterogeneous Co‐CMP catalysts, reactions were carried out at higher temperatures and CO_2_ pressures (100 °C, 3.0 MPa). Even under these intensified conditions, Co‐CMP displayed superior catalytic activity compared to KI (3.0%) and KI/β‐CD (13.2%) in the conversion of PO into PC, with yields of 98.1%. Co‐CMP also exhibited effectiveness in large‐scale catalysis over 60 h at 3.5 MPa CO_2_ and 130 °C, resulting in a PO TON of 40 660 (TON of values for KI and KI/β‐CD are 6 and 27, respectively, under similar conditions). Notably, the Co‐CMP catalyst could be recovered and reused up to 22 times without any significant decline in catalytic activity under the mentioned conditions. However, it was observed that the cobalt content of Co‐CMP slightly decreased after 11 cycles at room temperature and atmospheric pressure, and experienced a dramatic decrease after 22 recycling attempts. These findings suggest that prolonged exposure to the reaction solution could result in leaching from the cobalt sites in Co‐CMP.

In an extension of their earlier work, the research group developed Co‐CMP‐2, a compound capable of capturing and converting CO_2_ into cyclic carbonates under room temperature and atmospheric pressure conditions in 2017.^[^ ^80^ ^]^ This new catalyst showed significant improvements compared to Co‐CMP in the catalytic formation of PC from PO. Co‐CMP‐2 achieved a TON value of 236 and a yield of 98.7%, surpassing the previous reported values for Co‐CMP under similar conditions (TON 201, 98.1% yield). Furthermore, Co‐CMP‐2 demonstrated excellent reusability, as it could be utilized for more than 10 cycles without any noticeable decline in catalytic activity. No leaching of cobalt species was reported under these conditions.

The Su research group recently synthesized a new Zn‐salen‐based CMP (CMP‐Salen‐Zn) by the polycondensation of a dialdehyde derivative of salen‐Zn and pyrrole (Figure 2).^[^ ^87^ ^]^ PO was converted to PC at 99% yield after 12 h at 120 °C and 0.1 MPa CO_2_. The very good catalytic performance is likely owing to the accessible active sites of the CMP.

##### Porphyrin‐Based CMPs

The porphyrin unit also offers an opportunity for metalation with different catalytically active centers. Lu et al. developed a bifunctional catalyst (CPBr) that contains a Zn‐porphyrin as a Lewis acid (Figure 2), and quaternary phosphonium bromide salts (as nucleophilic reagents) to enhance the efficiency of the cycloaddition reaction through cooperative action.^[^ ^84^ ^]^ This catalytic system follows the promising advanced “Path B” route (Scheme 2), which eliminates the need for any additives (i.e., no co‐catalyst). The catalytic properties of CPBr‐2 were tested on the model substrate, PO, with 95% conversion to PC after 7 h at 90 °C and 2.5 MPa CO_2_ (Table 1, Entry 16).

An Al‐porphyrin‐based heterogeneous catalyst (Al‐iPOP) for the synthesis of cyclic carbonates from various epoxides and CO_2_ was reported by Chen et al.^[^ ^81^ ^]^ Al‐iPOP has both an active metal center and halogen anion, making it a bifunctional catalyst for additive‐free CO_2_ conversion. To enable industrial‐scale production, it is important for the catalysts to be able to efficiently catalyze the cycloaddition of epoxides with dilute CO_2_ under ambient conditions, especially since flue gases typically contain only 15% CO_2_ along with significant amounts of N_2_. It is noteworthy that Al‐iPOP is able to catalyze the cycloaddition reaction efficiently, even when using simulated flue gas (15% CO_2_ in N_2_, v/v) as feedstock, indicating its potential application in the utilization of CO_2_ emissions from industrial processes. Al‐iPOP showed excellent catalytic activity with a yield of 99% achieved after 7 h at 40 °C and 3.0 MPa. Additionally, a high TOF of 7600 h^−1^ was achieved for PO under reaction conditions of 100 °C and 1.0 MPa in 4 h. The authors attribute the catalytic activity of their materials to the intramolecular cooperative effect between the metal active center and the nucleophile, as well as the ability of the CO_2_‐philic catalysts to swell in the presence of substrates. The Al‐based bifunctional catalysts were designed to function based on the confinement effect, with the use of larger substrates leading to decreased yields due to slower diffusion rates through the narrow micropores (essentially promoting the path A route, Scheme 2).

Motivated by the fact that aluminum is the most abundant metal, Zhang et al. conducted research on another Al‐porphyrin‐based catalyst for the conversion of CO_2_ into cyclic carbonates.^[^ ^10^ ^]^ Al‐Por‐POP together with TBAB (Figure 1) formed PC (95.4%) with a high TOF of 65.5 h^−1^ under mild reaction conditions (48 h, 25 °C, 0.1 MPa, Table 1, Entry 24). Additionally, it should be noted that Al‐Por‐POP still exhibited a yield of 39.4% and TOF of 27 h^−1^ when using simulated flue gas and thus relatively low CO_2_ concentrations under these conditions.

In a further study by the same authors, they showed that ultrathin CMP nanosheets formed from porphyrin‐like structures (Figure 2) with single cobalt active sites act as heterogeneous catalysts for CO_2_ cycloaddition with various epoxides.^[^ ^85^ ^]^ These catalysts exhibited exceptional activity and stability under mild reaction conditions. At ambient temperature and pressure, PC has a yield of 99% and a TON value of 2903 (Table 1, Entry 18). The CMP nanosheets exhibit excellent catalytic stability owing to the strong coordination between the single‐site cobalt center and the nearest‐neighbor nitrogen atoms in the building units. The authors suggest that future research focusing on constructing CMP nanosheets with fewer layers, or even single layer, could further enhance catalytic efficiency by making more active sites accessible.

It is worth noting that the high surface areas in CMPs do not necessarily correlate with the highest catalytic activities. To illustrate, a unique Co‐porphyrin‐based microporous organic polymer (HUST‐1‐Co) was developed by Wang and colleagues.^[^ ^78^ ^]^ This material exhibits a hierarchical pore structure and abundant Co^2+^ ions, resulting in enhanced interaction between the pore walls and CO_2_. As a result, HUST‐1‐Co demonstrates a surface area of 1360 m^2^ g^−1^ and an impressive CO_2_ uptake of 21.39 wt.% at 273 K. However, despite these favorable attributes, the conversion to PC using HUST‐1‐Co was 95% (after 30 h at 25 °C and 0.1 MPa), which is lower than the 99% achieved by many other CMPs that possess significantly lower surface areas and CO_2_ uptakes (see Table 1, Entry 8).

TBB‐BPY‐Co, is another example of a Co‐coordinated CMP synthesized by Zhang's group. TBB‐BPY‐Co showed a surface area of 1279 m^2^ g^−1^ and CO_2_ uptake of 15.73 wt.% (273 K). However, conversion to PC was reported to only be 84.7%. It is worth noting that this reaction was conducted under mild conditions (25 °C and 1 bar), as shown in Table 1, Entry 21.^[^ ^86^ ^]^

It's noteworthy that recent studies have placed a spotlight on semi‐conjugated metalloporphyrin‐based polymers as catalysts for the conversion of CO_2_ into cyclic carbonates. Although outside the scope of this review, as they are not fully conjugated, we point the reader to several references. For instance, one well‐established example is the bifunctional AlPor−PIP−Br,^[^ ^94^ ^]^ which has demonstrated exceptional yield (98%) without requiring any co‐catalyst. This underscores the significance of the precisely matched spatial arrangement of aluminum centers and nucleophilic sites within the catalyst. Additionally, the same research group showcased the capabilities of AlPor‐iPAFs,^[^ ^95^ ^]^ where the integration of aluminum centers and halogen anions into the porphyrin networks has resulted in nearly quantitative yields of up to 99%, all achieved without the need for co‐catalysts or solvents. These results showcase the efficiency with which the synergistic effects can be finely tuned at the molecular level. A further illustrative example of the synergistic interplay between metal sites and nucleophilic anions is provided by Bai et al.^[^ ^96^ ^]^ They propose that the excellent catalytic performance of these catalysts can be attributed to three key factors: 1) a distinct spatial arrangement of active Lewis acidic sites and nucleophilic anions within metalloporphyrin‐based polymers to enhance the synergistic effects during the ring‐opening process; 2) a hierarchical nanoporous structure with a substantial surface area, facilitating the rapid diffusion of reactants and products; and 3) that the synthesized metalloporphyrin‐based polymers exhibit favorable swelling properties, which promote the entry of large reactant molecules into the catalytic sites within the polymers. This swellability not only enhances their catalytic activity but also improves their efficiency in the reaction.

##### Pyridine‐Based CMPs

Apart from salens and porphyrins, pyridines (Figure 2) possess a strong affinity for metal binding, making them suitable for the preparation of metalated monomers. Wu et al. synthesized pyridinic nitrogen‐functionalized porous organic polymers combined with CoCl_2_ (Co@PDVB‐VP‐0.5) with hierarchical pores (the pore sizes of these samples show very wide distributions ranging from 0 to 250 nm, confirming the micro‐meso‐macropore hierarchy formed).^[^ ^79^ ^]^ Pyridinic nitrogen atoms, among various nitrogen species, also exhibit interaction with CO_2_, making them potential sites for CO_2_ interaction. Co@PDVB‐VP‐0.5 has a surface area of 479 m^2^ g^−1^ and demonstrates efficient catalytic activity in converting CO_2_ from simulated flue gas into cyclic carbonates under ambient conditions (99.2% conversion to PC after 48 h, at 30 °C and CO_2_/N_2_ (0.15/0.85, 1.0 MPa)). Additionally, when the same reaction was conducted at 100 °C for 0.75 h, Co@PDVB‐VP‐0.5 achieved a PC yield of 98.9% and a TOF of 1034 h^−1^, indicating that the catalytic activity significantly increases at higher temperatures (Table 1, Entry 9). This enhanced catalytic activity for this class of materials can be attributed to the presence of hierarchical pores, the pyridinic nitrogen functionality, and the homogeneous dispersion of active metal sites.

#### 2.2.2 Metal‐Free CO_2_ Cycloaddition Reactions

Despite the significant advances in synthesizing metalated CMPs for the cycloaddition of epoxides with CO_2_, the incorporation of metals presents numerous obstacles. These challenges include metal leaching, deactivation of active metal sites through complexation or ionization, high expenses, and non‐sustainable practices. Consequently, there is a welcome focus on the development of metal‐free CMP‐based catalysts that exhibit exceptional catalytic performance and stability. This pursuit aims to establish innovative and sustainable pathways for utilizing CO_2_, as depicted in Scheme 2, Path B.

**Figure 3:**
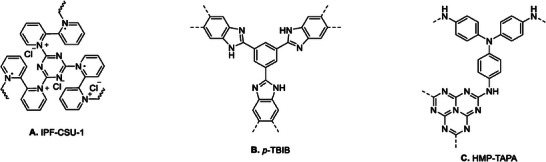
Metal‐free CMPs for catalysis of cycloaddition of CO_2_ to epoxides.

Yu et al. developed a porous framework called IPF‐CSU‐1 (**Figure** **3**A), which demonstrated favorable results for a metal‐free method (in the presence of TBAB as a co‐catalyst) of producing cyclic carbonates from CO_2_. The yield of this process was nearly quantitative (> 95%) at 25 °C and ambient pressure (Table 1, Entry 14).^[^ ^83^ ^]^ This success can be attributed to the combination of ionic functionalities and N‐rich components within the organic porous framework. However, it is important to note that the yields of the cycloaddition product decreased to 72% for PO at 100 °C, 12 h, and 0.1 MPa in the absence of TBAB (Figure 1), which serves as a co‐catalyst.

p‐TBIB (Figure 3B) represents another instance of a metal‐free binary catalyst. Ding et al. successfully synthesized this catalyst, consisting of microporous polymeric spheres.^[^ ^72^ ^]^ They employed p‐TBIB, together with the co‐catalyst TBAB, for the cycloaddition reaction of CO_2_ to generate organic cyclic carbonates. The authors propose that the nanopores of the CMP contain N‐doped CO_2_‐philic sites that contribute to the enhanced CO_2_ conversion (97%, 0.1 MPa, 25 °C, Table 1, Entry 15). The presence of amino‐containing electrophiles facilitates the ring opening of PO through hydrogen‐bonding interactions with the epoxide (see Scheme 2 (i‐iii)). Additionally, p‐TBIB displayed satisfactory catalytic performance even when the cycloaddition was conducted at low CO_2_ concentration resembling simulated flue gas (51% PC conversion; reaction conditions: TBAB co‐catalyst, 48 h, 298 K, and 1 bar).

Sharma et al. recently introduced a microporous polymer HMP‐TAPA that is rich in nitrogen and contains heptazine units (Figure 3C).^[^ ^68^ ^]^ The researchers investigated the cycloaddition of CO_2_ and terminal epoxides in the presence of TBAB, and observed that HMP‐TAPA exhibited high catalytic activity, leading to a cyclic carbonate product with a conversion rate of 99% (reaction conditions: 6 h, 80 °C, 0.6 MPa; Table 1, Entry 20). The catalytic activity of HMP‐TAPA can be attributed to the abundance of basic nitrogen sites, which facilitate selective capture and conversion of CO_2_.

**Figure 4:**
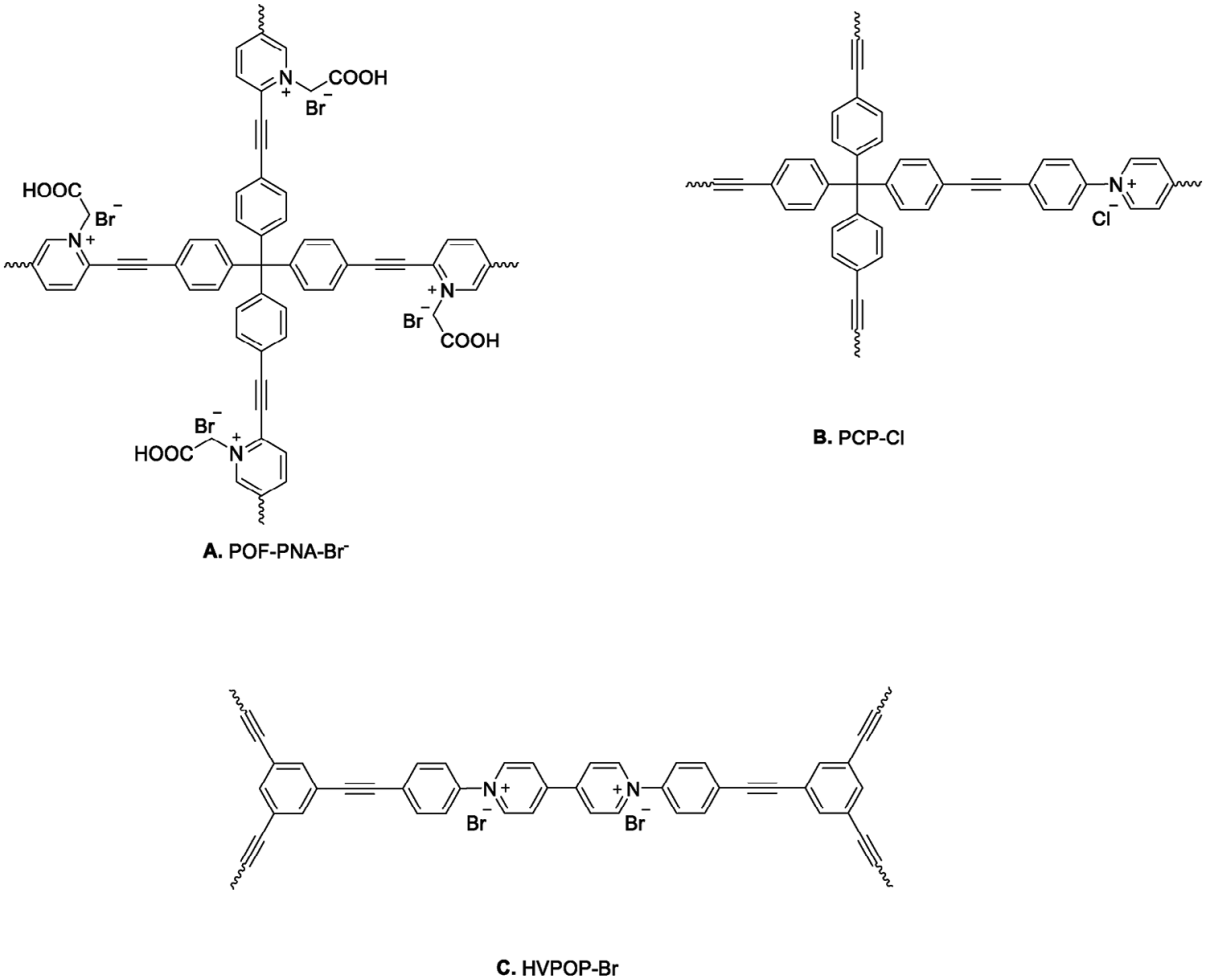
Metal and co‐catalyst free CMPs for catalysis of cycloaddition of CO_2_ to epoxides.

Ma et al. developed a novel metal‐free catalytic material by utilizing pyridine‐linkers and carboxylic acid groups through a post‐synthesis modification process.^[^ ^82^ ^]^ The resulting material, POF‐PNA‐Br^−^ (**Figure** **4**A), demonstrates the Brønsted acidic properties of the carboxylic acid group (‐COOH), while the coordinated Br^−^ anion associated with the pyridine linker acts as a nucleophilic active site. Owing to the cooperative effect of these two functional sites, POF‐PNA‐Br^−^ displays significant catalytic efficiency in the cycloaddition reaction of CO_2_ and PO, leading to the formation of PC with 98% yield. Notably, this reaction takes place under mild conditions (40 °C, 0.1 MPa, 48 h) and does not require any additional co‐catalysts (Table 1, Entry 13).

Buyukcakir and colleagues reported a further instance of a system that operates without the need for metal or co‐catalysts.^[^ ^77^ ^]^ The researchers described the synthesis of a charged porous polymer PCP‐Cl (Figure 4B), which exhibited good CO_2_ uptake (10.14 wt.% at 273 K) and catalyzed the formation of cyclic carbonates using epoxides and CO_2_. Among the various catalysts investigated, PCP‐Cl demonstrated the best performance (99% conversion to PC) under the reaction conditions of 100 °C, 3 MPa, and 12 h (Table 1, Entry 7). This performance can be attributed to the high nucleophilicity and the ability of Cl^−^ to serve as a leaving group.

As previously mentioned, nitrogen‐rich catalysts have shown great promise in facilitating the catalysis of CO_2_ cycloaddition reactions. In their recent research, Luo et al.^[^ ^89^ ^]^ engaged with this challenge by introducing viologen groups into their system. This strategic addition served a dual purpose: creating a nitrogen‐rich environment and forming the foundation of an ionic polymer backbone. The team designed and synthesized an ion‐exchanged, viologen‐based porous organic polymer featuring a hollow structure. This innovative approach demonstrated remarkable improvements in the catalytic cycloaddition of CO_2_, all within a metal‐free system and without the need for a co‐catalyst. Specifically, HVPOP‐Br (Figure 4C) emerged as a standout performer, showcasing excellent catalytic activity across a range of aliphatic and aromatic substrates (Table 1, Entry 25 and **Table** **2**, Entry 23) at reaction conditions of 120 °C, 1 MPa, and 12 h. This success underscores the critical roles played by the hollow structure and ion‐exchange mechanisms in viologen‐based POPs. Throughout the catalytic process, the Br^−^ ions residing within the pores assumed the role of nucleophiles, while the bipyridinium structure provided an electrophilic environment. This synergy between structure and ion properties highlights the importance of careful materials design to ensure the high performance of HVPOP‐Br in CO_2_ cycloaddition reactions.

**Table 2:** The catalytic performance of various materials for the chemical conversion of CO_2_ using different epoxides.

Entry	Polymeric material	Epoxide	*t* [h]	*T* [°C]	*P* CO_2_ [MPa]	Yield [%]	TOF [h^−1^]	TON	References
1	Zn‐CMP^a)^	Epichlorohydrin 1,2‐Butylene Oxide 1,2‐epoxyhexane Styrene oxide Glycidol 1,2‐epoxydodecane 1,2‐epoxy‐5‐hexene 2‐((ethynyloxy)methyl)oxirane Phenyl glycidyl ether 1,4‐Di(oxiran‐2‐yl)butane Isobutylene oxide 2,3‐Butylene oxide Cyclohexene oxide Stilbene oxide	1 1 1 1 2 2 2 2 2 2 12 24 12 40	120	3	99.6 96.4 96.1 96.4 91.2 80.6 94.1 95.6 92.6 93.1 90.6 93.5 66.3 56.1	n.r.	n.r.	[64]
2	Cr‐CMP^a)^	Epichlorohydrin 1,2‐Butylene Oxide 1,2‐epoxyhexane Styrene oxide	2	100	3	99.1 96.1 96.7 96.3	113 109 110 109	226 218 220 218	[74]
3	Zn@SBMMP^a)^	Styrene oxide Epichlorohydrin Phenyl glycidyl ether Butyl glycidyl ether Glycidol Allyl glycidyl ether	4	80	2	97 94 93 87 83 95	51 50 49.5 46.25 44.25 50.75	204 200 198 185 177 203	[75]
4	PCP‐Cl	Epichlorohydrin 1,2‐epoxyhexane Styrene oxide (Phenylmethyl)oxirane	12	100	3	98 85 16 41	n.r.	n.r.	[77]
5	HUST‐1‐Co^a)^	1,2‐Butylene Oxide Epichlorohydrin Epibromohydrin Styrene oxide	30 48 48 48	25	0.1	96.2 96.7 94.7 93.2	111.7 55.23 51.79 57.50	3350 2651 2486 2760	[78]
6	Co‐CMP‐2^a)^	Ethylene oxide	1	100	3	99.2	990	990	[80]
7	Al‐iPOP‐1 (Al‐iPOP‐2)	1,2‐Butylene Oxide Epichlorohydrin Allyl glycidyl ether 1,2‐epoxyoctane 1,2‐epoxydodecane Styrene oxide Cyclohexene oxide	6 6 9 9 9 9 36	40	1	99 (99) 99 (99) 51 (58) 40 (50) 8 (14) 52 (43) 83 (72)	n.r.	n.r.	[81]
8	POF‐PNA‐Br^−^	1,2‐Butylene Oxide Epichlorohydrin 1,2‐epoxyhexane Allyl glycidyl ether Styrene oxide	48	40	0.1	91.7 94.1 81.2 77.1 52.4	n.r.	n.r.	[82]
9	IPF‐CSU‐1^a)^	Epibromohydrin Epichlorohydrin	48	25	0.1	97 95	n.r.	n.r.	[83]
10	ZnTAPP‐Go‐r^a)^	Styrene oxide Phenyl glycidyl ether 1,2‐epoxyhexane 1,2‐epoxyoctane Cyclohexene oxide	10	100	0.1	88 99 95 88 34	n.r.	n.r.	[97]
11	p‐TBIB^a)^	Ethylene oxide 1,2‐Butylene Oxide Epichlorohydrin Styrene oxide Cyclohexene oxide Butyl glycidyl ether	24 24 24 96 96 96	25	0.1	95 93 89 81 73 66	9.675 9.467 9.058 2.060 1.858 1.679	232.2 227.2 217.4 197.8 178.4 161.2	[72]
12	CPBr‐2	Phenyl glycidyl ether Epichlorohydrin Styrene oxide	5 7 7	90	2.5	91 93 79	n.r.	n.r.	[84]
13	SCHPP‐3^a)^	Styrene oxide Epichlorohydrin Phenyl glycidyl ether m‐Tolylglycidyl ether Allyl glycidyl ether	1 5 5 5 5	120	2	74 >99 85 98 >99	n.r.	n.r.	[71]
14	DTBBQ‐CMP^a)^	Styrene oxide Phenyl glycidyl ether 4‐Tert‐butylphenyl glycidyl ether Butyl glycidyl ether 2‐Ethylhexyl glycidyl ether	48 48 72 48 72	60 50 60 50 65	0.1	99 99 99 99 99	35.71 30.23 13.58 28.65 12.90	1714 1451 978 1375 929	[85]
15	Zn‐saleN─CMP^a)^	1,2‐Butylene Oxide Epichlorohydrin Glycidol Cyclohexene oxide Styrene oxide	1	120	3	93 89 67 97 96	465 445 335 458 480	465 445 335 458 480	[70]
16	Co‐PPOP^a)^ (styrene oxide)	Styrene oxide Epibromohydrin Epichlorohydrin 1,2‐epoxyhexane	48 36 36 36	25	0.1	98 96 97 92	212.7 277.8 280.7 266.2	10 208 10 000 10 104 9583	[98]
17	HMP‐TAPA^a)^	Isobutylene oxide Epichlorohydrin 1,2‐Butylene Oxide 1,2‐epoxyhexane 1,2‐epoxydecane Butyl glycidyl ether Allyl glycidyl ether Styrene oxide Phenyl glycidyl ether	6	80	0.6	>98 >99 97 81 61 71 67 81 98^b)^ 57 98^c)^	77.8 78.7 77 64.3 48.4 56.3 53.1 64.3 45.1	467 472 462 386 291 338 319 386 467^b)^ 271 467^c)^	[68]
18	TBB‐BPY‐Co^a)^	1,2‐Butylene Oxide Epichlorohydrin Epibromohydrin Styrene oxide	48	25	1	80.3 83.6 82.5 66.7	290 302 298 241	13 918 14 490 14 299 11561	[86]
19	CMP‐Salen‐Zn^a)^	Epibromohydrin Epichlorohydrin Glycidol 1,2‐Butylene Oxide Cyclohexene oxide Styrene oxide	12	120	0.1	99 95 97 99 99 94	n.r.	n.r.	[87]
20	Co‐Por‐POP‐2^a)^	1,2‐Butylene Oxide Epichlorohydrin Epibromohydrin Styrene oxide Cyclohexene oxide	48	25	0.1	68.6 74.2 70.1 51.8 13.1	19.7 21.3 20.1 14.8 3.8	943.3 1020.3 964.0 712.3 180.1	[88]
21	Al‐Por‐POP‐2^a)^	1,2‐Butylene Oxide Epichlorohydrin Epibromohydrin Glycidol Butadiene monoxide Styrene oxide m‐Tolylglycidyl ether Cyclohexene oxide	48	25	0.1	91.5 96.2 92.9 80.1 85.5 72.8 83.6 22.8	62.8 66.0 63.7 55.0 58.7 50.0 57.4 15.6	3014 3168 3060 2638 2816 2398 2753 751	[10]
22	pPI‐1/pPI‐2	Epichlorohydrin Epibromohydrin	72	80	0.1	98/90 89/72	n.r.	n.r.	[73]
23	HVPOP‐Br	Epichlorohydrin Styrene oxide Phenyl glycidyl ether o‐tolyl glycidyl ether Glycidol Allyl glycidyl ether	12	120	1	98 85 96 94 93 76	n.r.	n.r.	[89]

^a)^ TBAB co‐catalyst;

^b)^ Reaction time 12 h;

^c)^ reaction time 15 h.

#### 2.2.3 Other Epoxide Conversions

**Figure 5:**
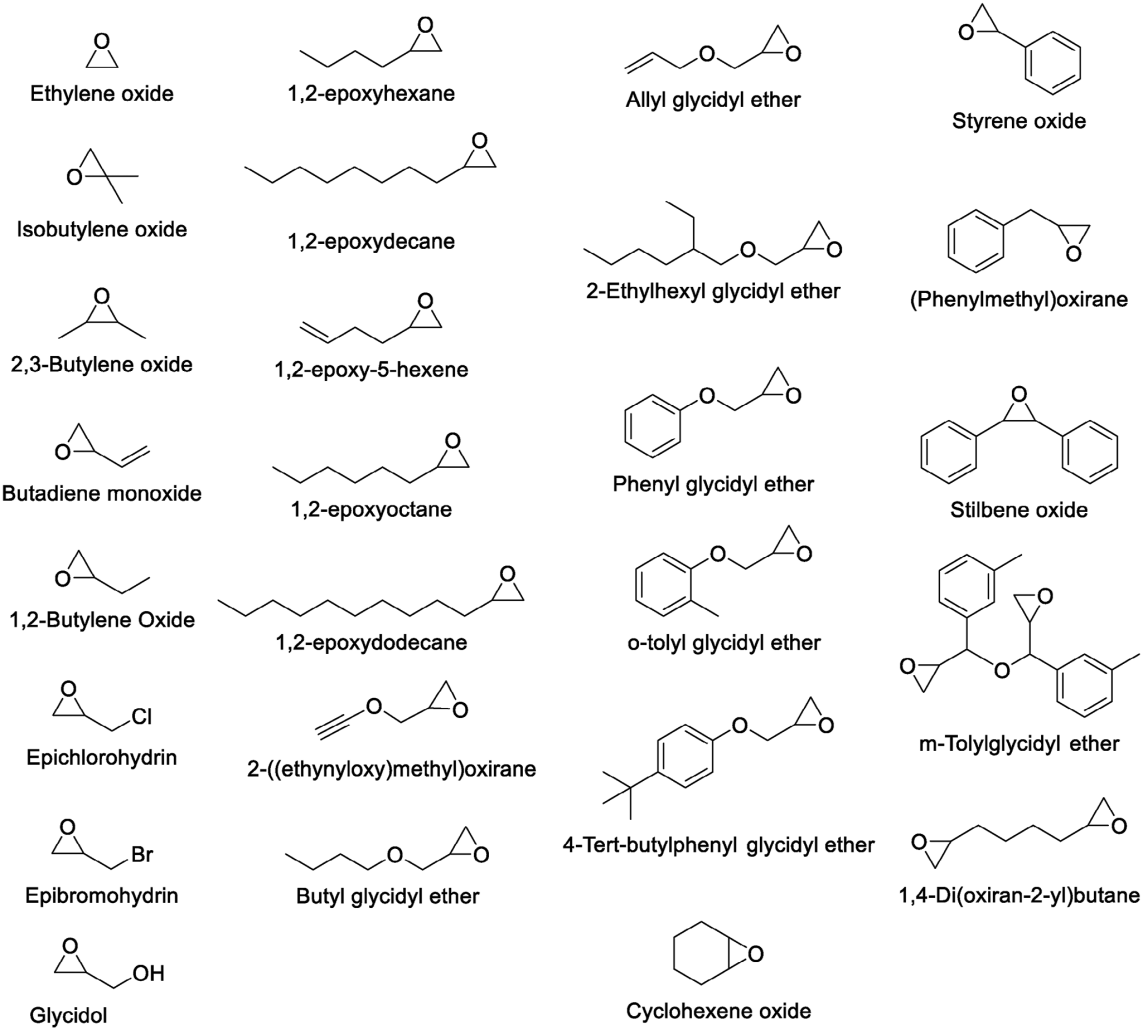
Structures of the various epoxides explored for cycloaddition with CO_2_.

Apart from the model PO conversions discussed previously in Sections 2.2.1. and 2.2.2. other substrates including further terminal epoxides, challenging internal epoxides and aromatic epoxides are also covered in this review (see **Figure** **5**). Many of both the metal‐ and non‐metal‐based CMPs detailed previously show high conversion yields (above 90%) for other epoxides (see Table 2 for details).

In most of the studies discussed previously, the catalytically active materials also demonstrated good conversion of styrene oxide to styrene carbonate (see Table 2). Of particular note are the three metal‐based catalytic systems Zn@SBMMP (salen‐based), DTBBQ‐CMP (tetra amino phthalocyanine‐based) and Co‐PPOP (porphyrin‐based), each reliant on a TBAB co‐catalyst. Interestingly DTBBQ‐CMP has the lowest surface area of the three (16 m^2^ g^−1^, see Table 1, Entry 18) yet produces the highest conversion yield (99%). Of the three, those with cobalt as the active metal produced high conversion yields at lower temperatures and pressures (see Table 2 for details).

Internal epoxides, such as cyclohexene oxide are particularly hard to convert due to steric hindrance of the starting material, with many catalyzed reactions giving low conversion yields. However, there are some salen‐ and zinc‐based porous catalysts that have achieved excellent yields of up to 99% for the synthesis of the corresponding cyclohexene carbonate (see Table 2).

**Figure 6:**
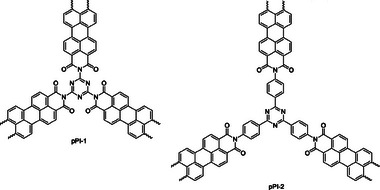
Structures of metal and co‐catalyst free pPIs for catalysis of cycloaddition of CO_2_ to halogenated epoxides.

Finally, with respect to halogenated epoxides, our group has recently synthesized two perylene‐based porous polyimides (pPIs) (**Figure** **6**). These porous materials, based on perylene‐3,4,9,10‐ tetracarboxylic dianhydride and melamine in the case of pPI‐1 and tris‐(4‐aminophenyl)triazine in the case of pPI‐2, respectively, were synthesized in a catalyst‐free polycondensation reaction.^[^ ^73^ ^]^ These materials exhibited excellent metal‐ and co‐catalyst‐free catalytic performance in the synthesis of cyclic carbonates from CO_2_ and halogenated epoxides. High yields of up to 98% was achieved under very mild conditions (353 K, 1 bar CO_2_) and in the absence of solvents. pPIs thus present a promising metal‐free, green and sustainable solution for the fixation of CO_2_ into useful fuels and chemical feedstocks.

Although the conversion of epoxides to cyclic carbonates represent a promising field for CO_2_ capture and conversion, the maximal market for cyclic carbonates is ≈100 kt pa.^[^ ^99^ ^]^ As detected CO_2_ emissions for 2023 are approaching ≈40 billion metric tons,^[^ ^100^ ^]^ it is crucial to consider other routes to CO_2_ capture and conversion, as discussed below.

### 2.3 Other Routes for the Chemical Conversion of CO_2_

**Scheme 4:**
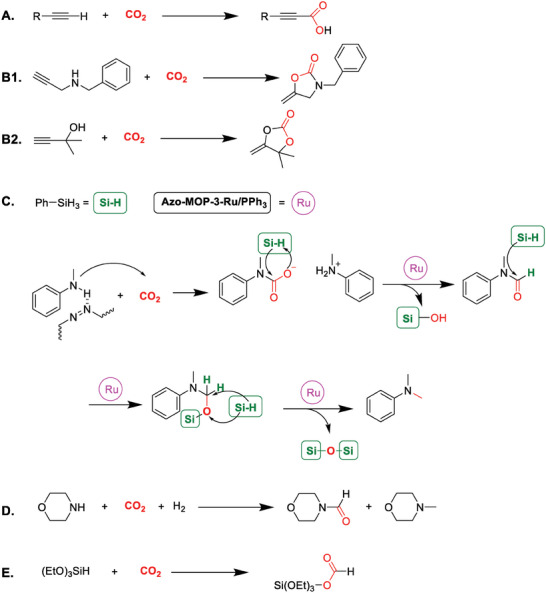
Reaction pathways to CO_2_ conversion via alternative non‐epoxide routes catalyzed by CMPs.

In addition to the commonly used cycloaddition reaction of CO_2_ with epoxides, there are several other reactions that utilize CO_2_ as a starting material and are catalyzed by CMPs. These reactions, including carboxylation,^[^ ^12^ ^]^ methylation,^[^ ^13^ ^]^ and hydrosilylation^[^ ^101^ ^]^ form a variety of products, as detailed in **Table** **3** and **Scheme** **4**.

**Table 3:** Non‐epoxide routes and yields for the chemical conversion of CO_2_ catalyzed by CMPs.

Entry	Route of conversion	Polymeric material	BET [m^2^g^−1^]	CO_2_ uptake [wt%]	*t* [h]	*T* [°C]	*P* CO_2_ [MPa]	Yield [%]	References
1	A. Carboxylation of alkynes	CMP‐Cu	–	–	60	25	0.1	31.2	[12]
2		MOP‐PZ−Ag	897.5	11.55^a)^ 18.37^b)^	24	50	0.1	92	[102]
3		Ag@NPOP‐1 Ag@NPOP‐2	–	–	12	60	0.1	94 92.1	[103]
4	B1. Carboxylative cyclization of propargyl amines	Ag@NPOP‐1 Ag@NPOP‐2	–	–	2	50	0.1	97 93	[103]
5	B2. Carboxylative cyclization of propargyl alcohols	Ag@UCPP	102	2.65^a)^ 2.92^b)^	24	25	1	99	[104]
6		F‐MOP‐3‐Ag	399	6.3^b)^	10	25	1	100	[105]
7	C. Methylation of amines	Azo‐MOP‐3‐Ru	370	8.21^b)^	24	120	0.5	99	[13]
8	D. Formylation of amines	CarPy‐CMP@Ru	735	9^a)^ 17.1^b)^	24	130	4	91	[11]
9	E. Hydrosilylation	CMP‐NHC‐CuCl	388	–	10	20	0.1	91.7	[101]

^a)^ 298 K;

^b)^ 273 K

**Figure 7:**
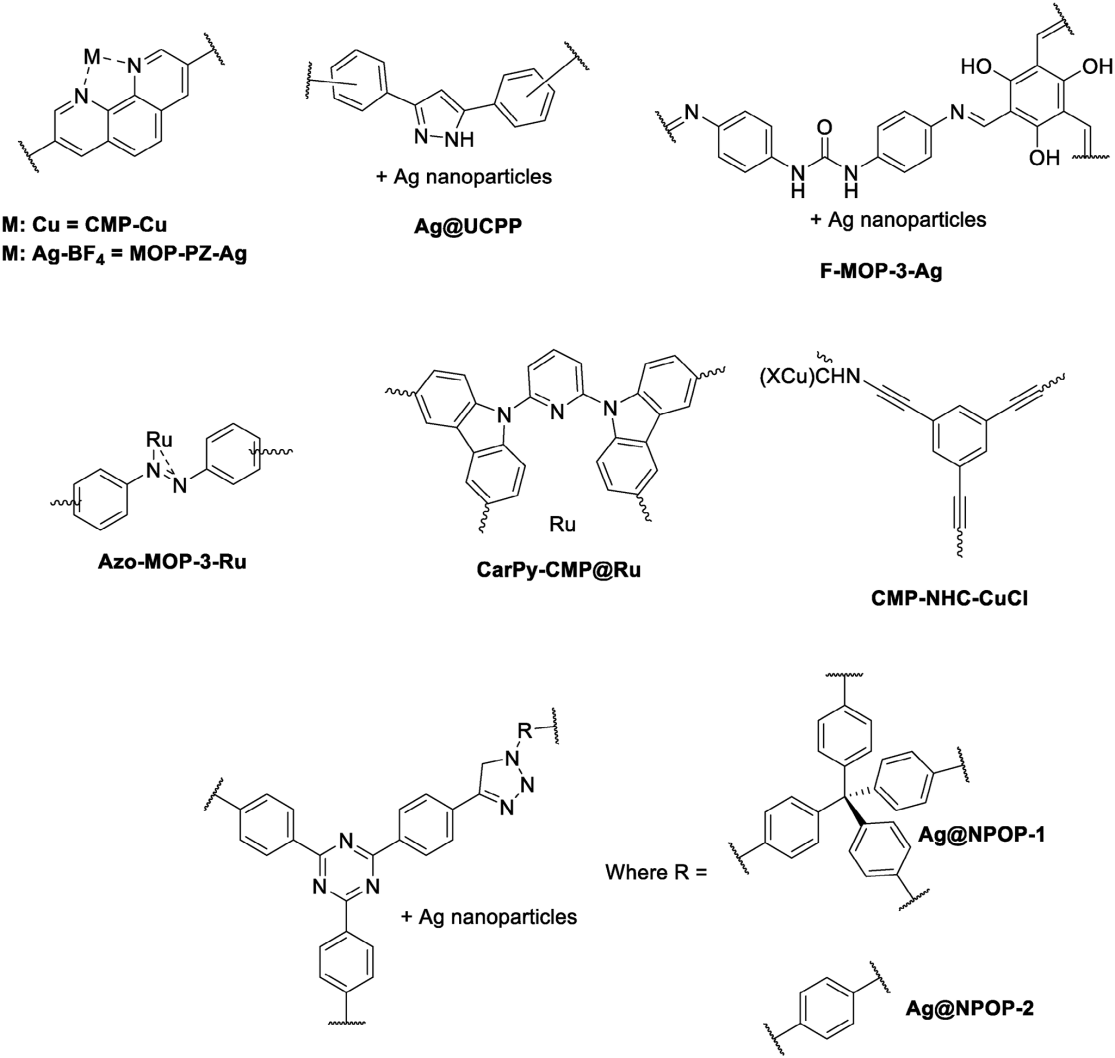
Structures of CMPs for other routes for chemical conversion of CO_2_.

Carboxylic acids (Scheme 4A), for example, have significant roles in both organic chemistry and industry, with a variety of applications in different areas, including in pharmaceuticals, agrochemicals, coatings, food and feed industry, cosmetics and materials for disinfection.^[^ ^106^, ^107^, ^108^, ^109^, ^110^, ^111^ ^]^ In addition to all these uses, carboxylic acids are essential building blocks for the production of derivatives such carboxylate salts,^[^ ^112^ ^]^ esters,^[^ ^113^ ^]^ nitriles,^[^ ^114^ ^]^ and amides.^[^ ^115^ ^]^ Synthesis of carboxylic acids from CO_2_ has been catalyzed by several CMPs. Xie et al. developed a copper‐based CMP (CMP‐Cu, see **Figure** **7**) and employed it for the direct C–C coupling between CO_2_ and terminal alkynes. This reaction occurred at room temperature and atmospheric pressure, yielding the corresponding carboxylic acids at a 31.2% yield (see Scheme 4A).^[^ ^12^ ^]^

Cui et al.^[^ ^102^ ^]^ reported another example of carboxylation of alkynes with CO_2_ using a porous polymer composite containing silver nanoparticles (MOP‐PZ−Ag, Figure 7). The catalytic process resulted in the production of alkynyl carboxylic acids with a high yield (92%) at 50 °C and ambient pressure (0.1 MPa). The catalyst exhibited recyclability for up to five cycles without significant degradation or loss of efficiency. Alkynyl carboxylic acids offer various advantages in terms of handling and storage when employed as a source of alkynes. It should be noted that the formation of alkynyl carboxylic acids presents a favorable alternative to terminal alkynes with low boiling points, as it stabilizes the alkynes and prevents dimerization. Moreover, the availability of commercially accessible alkynyl carboxylic acids is limited, thus novel approaches to synthesizing these compounds, such as the chemical conversion of CO_2_, have garnered significant interest.^[^ ^116^ ^]^

In a recent investigation carried out by Wu et al.,^[^ ^103^ ^]^ two materials were designed and synthesized that exhibited catalytic performance in both the carboxylation of terminal alkynes (Table 3, Entry 3, up to 94% yield) and the carboxylative cyclization of propargylic amines with CO_2_ with yields of up to 97% (Table 3, Entry 4; Scheme 4‐B1). The catalytic efficacy can be attributed to several factors: a) Ag@NPOPs incorporates two distinct types of nitrogen heterocycles, specifically triazine and triazole rings. These heterocycles possess the ability to capture and concentrate CO_2_ while also serving as anchoring points for Ag nanoparticles; b) NPOPs possess an abundance of micropores (the micropore volumes are 0.23 and 0.11 cm^3^ g^−1^ for NPOP‐1 and NPOP‐2, respectively) and a high specific surface area (481 and 233 m^2^ g^−1^ for NPOP‐1 and NPOP‐2, respectively), providing them with exceptional affinity for CO_2_ and significant adsorption capacity; c) the Ag nanoparticles within Ag@NPOPs are highly dispersed, which further enhances their catalytic efficiency. The Ag@NPOPs exhibit catalytic stability and durability, maintaining their catalytic activity without significant degradation over five consecutive cycles.

Terminal alkynes were also employed by Wang et al. to extend the range of substrates for CO_2_ conversion. The carboxylative cyclization of propargyl alcohols with CO_2_ (Scheme 4‐B2) is a clean and attractive process for synthesizing α‐alkylidene cyclic carbonates, crucial building blocks in organic and pharmaceutical syntheses.^[^ ^104^ ^]^ The Ag@UCPP catalyst they employed (see Figure 7) offers advantages such as enhanced interaction with CO_2_ molecules, thanks to the abundance of CO_2_‐philic groups, and stronger interaction with Ag nanoparticles. Importantly, the Ag@UCPP catalyst maintained high catalytic activity even after five cycles, demonstrating good recyclability and stability. They examined the carboxylative cyclization of CO_2_ with various alcohols containing an alkyne functional group under optimized conditions. All alcohols with both linear and cyclic alkane substituents were successfully converted into the desired α‐alkylidene cyclic carbonates with isolated yields over 90% (Table 3). This process, which involves the fixation of CO_2_ through the cycloaddition of propargylic alcohols, allows for the creation of five‐membered α‐alkylidene cyclic carbonates—an atom‐economic process with the products having widespread application as precursors in the synthesis of organic materials.^[^ ^117^ ^]^

Yang et al. also investigated the carboxylative cyclization of propargyl alcohols with CO_2_ (Scheme 4‐B2) under mild conditions.^[^ ^105^ ^]^ They designed a phenanthroline‐containing F‐MOP coordinated with Ag(I), which served as a dual catalyst for activating CO_2_ and propargyl alcohols simultaneously. This catalyst system exhibited high efficiency, resulting in 100% yields of α‐alkylidene cyclic carbonates under mild conditions (1 MPa, 25 °C) without the need for a co‐catalyst. The catalytic activity of F‐MOP‐3‐Ag with BF_4_ surpassed that of the base Ag without CMP owing to the even distribution of Ag within F‐MOP‐3‐Ag and the specific interaction between the fluorinated support and CO_2_ (see Figure 7). F‐MOP‐3‐Ag also demonstrated good reusability, as evidenced by its successful reuse five times. Importantly, the presence of F‐MOP‐3‐Ag enabled efficient reaction of both internal and terminal propargyl alcohols with CO_2_, resulting in 99% yields of the corresponding α‐alkylidene cyclic carbonates. It is worth noting that no catalytic activity was observed in the absence of the fluorinated additive.

The same research group also investigated the methylation of amines using CO_2_ (Scheme 4C) as a C1 source and Azo‐MOP‐N‐Ru as a catalyst.^[^ ^13^ ^]^ The formed methylamine products from Scheme 4C, are basic reagents in nitrogen chemistry and typically used as platform chemicals or solvents, and for the development of new drugs. Furthermore, CO_2_ has obvious advantages in the use of N‐methylation reactions to replace conventional hazardous methylation reagents such as diazomethane and methyl iodide.^[^ ^118^ ^]^ The Azo‐MOP‐N‐Ru catalysts (see Figure 7) demonstrated high yields (92‐99%) in the methylation of a range of amines with CO_2_ under low‐pressure conditions (120 °C; 0.5 MPa), encompassing a wide range of reactants. The performance of Azo‐MOP‐N‐Ru catalyst surpassed previously reported ruthenium‐based, non‐CMP homogeneous catalytic systems, which required higher CO_2_ pressures (e.g., 3 MPa) to achieve a comparable yield of 92%. Significantly, Azo‐MOP‐3‐Ru exhibited good reusability, a feature not observed in non‐CMP catalysts, as evidenced by obtaining a 95% yield even after five cycles of catalyst reuse. Additionally, Azo‐MOP‐3‐Ru proved effective in reactions involving various amines with CO_2_ under low‐pressure conditions (120 °C; 0.5 MPa). N‐methylanilines carrying both electron‐donating and electron‐withdrawing groups were successfully transformed into the corresponding N,N‐dimethylanilines with excellent yields (93‐99%). Moreover, N‐methylanilines substituted with chlorine or methyl groups at the ortho‐, meta‐, and para‐positions of the benzene ring were all efficiently converted into methylamines with high yields. Dialkylamines also exhibited good reactivity, achieving a yield of 92% when catalyzed by Azo‐MOP‐3‐Ru under similar conditions. Furthermore, Azo‐MOP‐3‐Ru demonstrated its catalytic capability in the reduction of formamide, leading to the preferential formation of N,N‐dimethylaniline with a yield of 99%.

Yang et al. further expanded their research into other routes for the chemical conversion of CO_2_ by studying formylation of amines with CO_2_/H_2_ (at equal pressures, total pressure of either 4 or 8 MPa) (Scheme 4D).^[^ ^11^ ^]^ Formamides, which are widely used as solvents and starting materials in organic synthesis, were targeted in this study. To achieve a more environmentally friendly approach for N‐formylation of amines, the researchers utilized CO_2_ and H_2_ as a formylating reagents. They employed a ruthenium‐coordinated CMP (CarPy‐CMP@Ru, see Figure 7) as a catalyst. The CMP exhibited uniform pore size distribution of ≈1.7 nm, a hierarchically porous structure, with a BET surface area of ≈1000 m^2^ g^−1^ and a very good CO_2_ uptake capacity of up to 17.1 wt.% at 1 bar and 273 K. CarPy‐CMP@Ru efficiently catalyzed the formylation of amines using CO_2_ and H_2_, resulting in high product yields ranging from 89% to 93%. The polymer not only served as a support for the catalytic Ru nanoparticles but also possessed the ability to capture CO_2_ due to the CO_2_‐philic nature of the pyridine functionality and hierarchical porosity. Furthermore, it activated amines through the formation of hydrogen bonds.

The reaction demonstrated that CarPy‐CMP@Ru catalyst was highly effective, resulting in a 91% yield of N‐formylmorpholine. This yield was significantly higher compared with the commercially available Ru/C catalyst (29% yield; 24 h reaction at 130 °C, and a pressure of 4 MPa). Importantly, the CarPy‐CMP@Ru catalyst exhibited excellent reusability and recyclability through filtration. This was confirmed by achieving a 91% yield even after the catalyst was reused five times. Additionally, when CO_2_/H_2_ was present, other cyclic secondary amines like 4‐methylpiperidine, pyrrolidine, and piperidine were converted to their corresponding formamides with yields ranging from 90% to 93% under the optimized conditions. Moreover, dialkylamines such as dipropylamine resulted in the corresponding formylation product with an 89% yield.

The final CMP‐involved catalytic pathway is the hydrosilylation of CO_2_ with triethoxysilane (Scheme 4, Reaction E), to yield silyl formate, a product that can be utilized for further transformation to yield, among other, carbonyl products. Compared with hydrogenating CO_2_ with H_2_, this reaction is a thermodynamically favorable process.^[^ ^119^ ^]^ CMP‐NHC‐CuCl (Figure 7), synthesized by Zhou et al., exhibited high efficiency in catalyzing the mild hydrosilylation of CO_2_ with triethoxysilane to produce silyl formate. The reaction conditions involved a temperature of 20 °C, pressure of 0.1 MPa, and a duration of 10 h, resulting in a yield of 91.7% (Table 3, Entry 9).^[^ ^101^ ^]^ This performance surpassed that of numerous metal‐based systems.^[^ ^120^, ^121^ ^]^

## 3 Electrochemical CO_2_ Reduction (ECO_2_R)

Electrochemical conversion is another promising way to recycle CO_2_ and create a sustainable circular carbon economy. This method involves reactions that produce reduced forms of CO_2_. For these reactions to occur, external energy input is needed. A major breakthrough in this area occurred in 1985, when Hori et al. discovered that various metal electrodes could reduce CO_2_ to formate, carbon monoxide (CO), and hydrocarbons like methane, under applied potentials.^[^ ^122^ ^]^ Since then, many researchers continue to explore better electrocatalysts be developing and optimizing better materials, computational methods, and characterization techniques.^[^ ^122^ ^]^

### 3.1 Fundamental Principles of the ECO_2_R

As experimental studies and theoretical simulation have revealed,^[^ ^123^, ^124^, ^125^ ^]^ the ECO_2_R process principally consists of 3 steps: CO_2_ activation, surface reaction and product desorption.

**Scheme 5:**
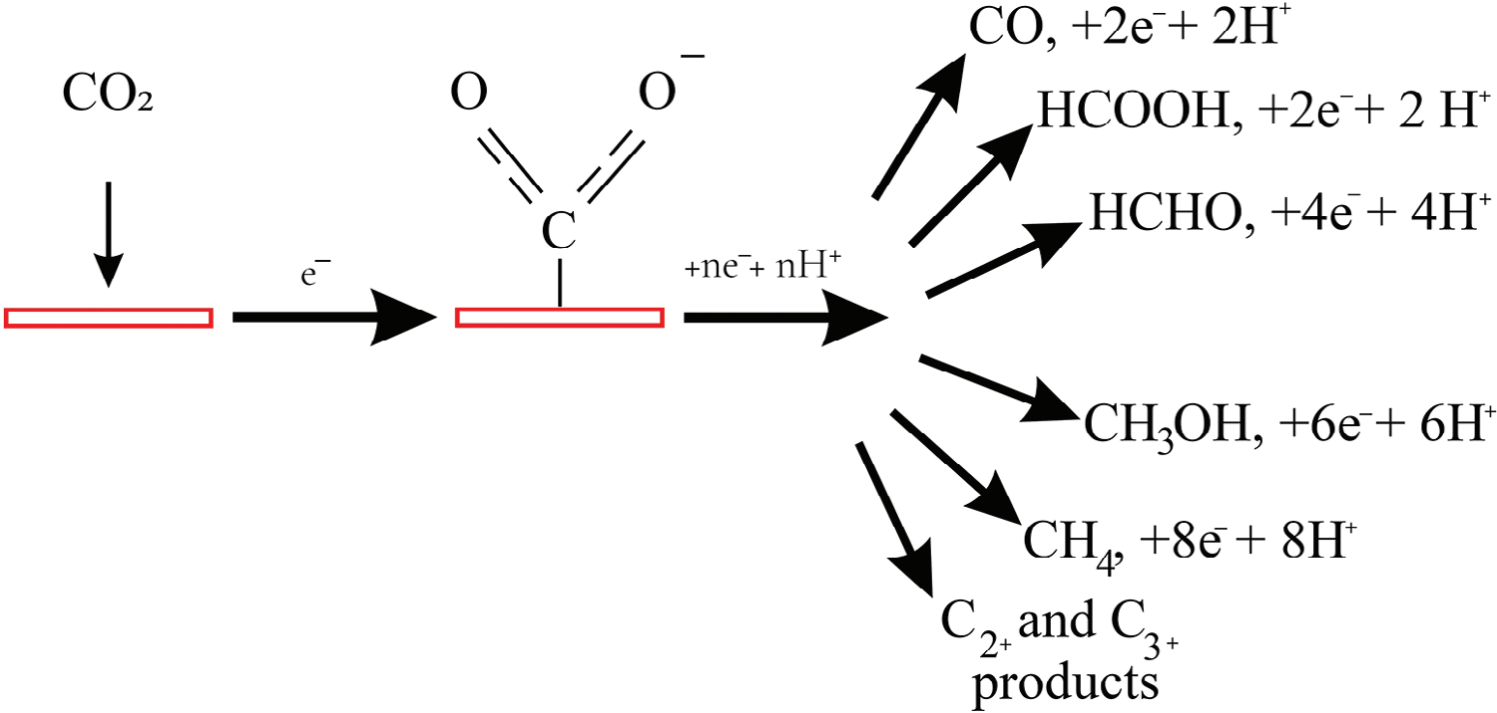
Showing activation of bent carbon dioxide on catalytic support and reduction to a variety of products.

Since CO_2_ is a very stable molecule with a high C═O dissociation energy of ≈750 kJ mol^−1^, breaking this bond to create new compounds is challenging.^[^ ^126^, ^127^ ^]^ In the activation step, bent configurations of CO_2_ will be formed by establishing chemical bonds between the linear CO_2_ structure and the catalyst, thus weakening the C═O bond (**Scheme** **5**). Moreover, the LUMO energy level decreases when the molecule bends, lowering the barrier for electron acceptance.^[^ ^127^ ^]^ Due to the solvent and internal rearrangement, the one‐electron reduction of CO_2_ to generate the bent CO_2_^·−^ species demands a high negative potential (E_0_ = −1.50 V vs RHE in aqueous media).^[^ ^128^ ^]^ Catalysts are required to solve the energy barrier problems related to this step, and for CO_2_ reduction to be effective and selective.^[^ ^129^ ^]^

An ideal catalyst should be designed to operate at a low overpotential with a high Faradaic efficiency (FE), current density (J), energy efficiency and stability. Only a brief overview of these characteristics will be provided here, as these topics are discussed in detail elsewhere.^[^ ^130^, ^131^, ^132^ ^]^

The difference in value between the applied potential and the equilibrium potential (the potential at which the concentration gradient of the electrolyte is balanced by the applied potential) required to drive electrocatalysis is known as the overpotential.

The proportion of electrons used to make a specific product is known as the FE. It can be found by dividing the required number of moles of electrons by the total number of electrons moved during the electrolysis process from the anode to the cathode. The following equation (Equation 1) is used to calculate the FE:

(1) FE=αnF/Q

where α represents the number of electrons moved, n is the number of moles of electrons for a particular product, F is Faraday's constant (96 485 C mol^−1^), and Q is the total amount of charge moving through the cell.^[^ ^133^ ^]^

When comparing how much energy the cathode and anode components of an electrochemical cell use overall, the total energy efficiency is crucial. Equation 2 can be used to calculate the energy efficiency:

(2) $$\mathrm{Energy}\;\mathrm{efficiency}=\Delta E^{0}/\Delta E_{\mathrm{applied}}*\mathrm{FE}$$

where ∆E^0^ is the theoretical equilibrium potential difference for the production of a particular product during ECO_2_R, while ∆E_applied_ is the system's actual applied voltage. FE is the product's Faradaic efficiency.^[^ ^133^ ^]^

The product formation rate (productivity) is presented by the current density (J) – the measure of electric current per unit area of the electrode at a given potential. Current density is related to the density of active sites and reaction kinetics.

The product formation rate, which may be calculated as the amount of product divided by the mass of catalyst and reaction time, is the most frequently used measure to assess the efficacy of a catalytic system. Accordingly, the rate is commonly expressed as mol of product per hour per gram of catalyst (Equation 3); however, the product can also be expressed in millimolar units (mmol) or as a concentration (ppm).^[^ ^127^ ^]^ Despite the fact that this rate is frequently employed in the literature to indicate catalytic activity, it should be emphasised that catalytic activity does not exhibit linear correlations with catalyst amount and reaction time, making it a less‐than‐ideal indicator.^[^ ^19^ ^]^

(3) $$\begin{array}{ccc} & \mathrm{Rate} & \;\mathrm{of}\;\mathrm{Production}\;\left(\mathrm{mol}/\left(\mathrm{hg}\right)\right)\\ & & =\mathrm{Amount}\;\mathrm{of}\;\mathrm{Product}\;\left(\mathrm{mol}\right)/\left(\mathrm{Mass}\;\mathrm{of}\;\mathrm{Catalyst}\;\left(g\right)*\mathrm{time}\;\left(h\right)\right) \end{array}$$

As an alternative, turnover frequency (TOF, Equation 4) is often regarded as the best metric to describe catalytic activity and allow for comparison of catalyst activities.^[^ ^19^, ^134^ ^]^ The majority of studies do not publish TOF data since it is challenging to estimate the quantity of active sites on a surface, which makes it challenging to compare various catalysts. Catalyst recyclability and stability, along with catalytic efficiency, are further important considerations for assessing catalysts and serve as key markers for prospective applications.^[^ ^19^ ^]^

(4) $$\mathrm{TOF}=n_{\mathrm{product}}/n_{\mathrm{site}}$$

where n_product_ is amount of product in moles and n_site_ is number of active sites on a surface.

In terms of catalyst stability, the problem of deactivation has frequently been discussed. The primary causes for loss of catalyst activity and deactivation are the production of intermediates that can bind to the catalyst (catalyst poisoning) or the deposition of inactive materials on electrode surfaces.^[^ ^135^ ^]^

An effective catalyst for ECO_2_R should have fast electron and mass transport as well as highly exposed active sites. In general, it is difficult to meet these conditions in a single catalyst, although a catalyst with a high surface area and porous architecture presents a potential solution. This design could be accomplished using a hierarchical porous morphology, owing to the following advantages: macropores can effectively reduce the ion diffusion distance by providing a high‐volume buffer for electrolyte ions; fast mass transportation via diffusion is possible with mesopores; and the specific surface area can be improved using micropores.^[^ ^136^, ^137^ ^]^

CO, formate, formaldehyde, methane, methanol, and C2+ hydrocarbons and oxygenates are only a few examples of the many conceivable carbon products that can be produced during the reduction of CO_2_, based on the multi‐electron stepwise reduction processes listed in **Table** **4**.^[^ ^138^, ^139^ ^]^ Thus, electron transfer impacts both the rate of CO_2_ reduction and the product selectivity.

**Table 4:** Equilibrium potentials of possible ECO_2_R reactions (RHE = reversible hydrogen electrode).^[^ ^140^ ^]^

Reactions	*E*° [V vs. RHE]
2H_2_O → O_2_ + 4H^+^ + 4e^−^	1.23
2H^+^ + 2e^−^ → H_2_	0
${\mathrm{CO}}_{2}+e^{-}\to {\mathrm{CO}}_{2}^{\cdot -}$	−1.50
CO_2_ + 2H^+^ + 2e^−^ → CO + H_2_O	−0.11
CO_2_ + 2H^+^ + 2e^−^ → HCOOH	−0.12
CO_2_ + 4H^+^ + 4e^−^ → HCHO + H_2_O	−0.07
CO_2_ + 6H^+^ + 6e^−^ → CH_3_OH + H_2_O	0.03
CO_2_ + 8H^+^ + 8e^−^ → CH_4_ + 2H_2_O	0.17
2CO_2_ + 8H^+^ + 8e^−^ → CH_3_COOH + 2H_2_O	0.11
2CO_2_ + 10H^+^ + 10e^−^ → CH_3_CHO + 3H_2_O	0.06
2CO_2_ + 12H^+^ + 12e^−^ → C_2_H_4_ + 4H_2_O	0.08
2CO_2_ + 12H^+^ + 12e^−^ → C_2_H_5_OH + 3H_2_O	0.09
2CO_2_ + 14H^+^ + 14e^−^ → C_2_H_6_ + 4H_2_O	0.14
3CO_2_ + 18H^+^ + 18e^−^ → C_3_H_7_OH + 5H_2_O	0.10

### 3.2 Factors Affecting ECO_2_R

For practical uses, the ability of a catalyst to selectively form desired compounds during ECO_2_R is crucial. This selectivity typically has a close relationship to the reduction mechanism, with various reaction pathways leading to various products. Experimental parameters, including CO_2_ concentration, type and concentration of electrolyte used as well as type and design of the electrolyser, can have a significant impact on the type and the number of pathways available for the reduction process,^[^ ^141^ ^]^ and are discussed below:

#### 3.2.1 Hydrophobicity of the Catalyst

**Figure 8:**
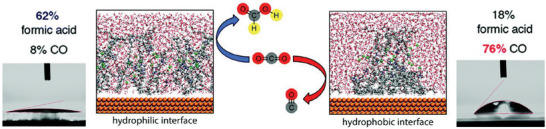
Wettability tests for Cu catalysts decorated with hydrophilic and hydrophobic organic modifiers leading to significantly different product distributions. Adapted with permission.^[^ ^142^ ^]^ Copyright 2019, American Chemical Society.

Proton and CO_2_ concentrations at the electrode surface have a significant impact on CO_2_ reduction, and both can be controlled by modifying the surface wettability. According to Buckley et al.,^[^ ^142^ ^]^ ECO_2_R selectivity for hydrogen (H_2_), CO and formic acid (HCOOH) could be tuned by using organic modifiers (usually polymer coatings) to change the surface wettability of Cu catalysts. Protic, hydrophilic, and hydrophobic species were found to improve the selectivity for the generation of H_2_, CO, and HCOOH, respectively. The selectivity is also influenced by the hydrocarbon concentration of the organic modifier compound: a higher hydrocarbon content increases the selectivity for CO, while a lower hydrocarbon content increases the selectivity for HCOOH (**Figure** **8**). These results indicate that the product selectivity is dependent on the wettability of the (metal) catalyst surface, opening up a new avenue for the development of ECO_2_R catalysts by carefully tailoring surface hydrophilicity.

#### 3.2.2 Electrolyte Choice

The most common inorganic electrolytes for the ECO_2_R are sodium and potassium bicarbonates (KHCO_3_), which are used as both proton donors and pH buffers.^[^ ^143^ ^]^ The precise function of bicarbonate solutions, however, is still up for debate. Wuttig et al. developed microkinetic models and presented a diagnostic criterion to identify the function of bicarbonate.^[^ ^144^ ^]^ They found that bicarbonate did, in fact, function as a useful proton donor. Additionally, bicarbonate served as a buffer by simply maintaining the pH of the system. Moreover, by combining mass spectroscopic methods, isotopic labeling, and surface‐enhanced IR absorption spectroscopy, the function of interfacial bicarbonate anions in the ECO_2_R was also investigated.^[^ ^145^ ^]^ It was discovered that bicarbonate might speed up the ECO_2_R reaction rate by raising the CO_2_ concentration via a quick equilibrium exchange with dissolved CO_2_. It was established that KHCO_3_ had a multi‐faceted role in the reaction, serving as both a pH buffer and a proton donor while simultaneously donating CO_2_ molecules to act as CO_2_ reactant.^[^ ^146^ ^]^ Additional research supported the notion that bicarbonate anions are the primary source of CO_2_ reduction products.^[^ ^147^ ^]^

#### 3.2.3 Apparatus Setup

An ideal electrolyzer would prevent electrolyte mixing between the anode and cathode compartments while promoting electron transfer, ion transport, and regulate gas diffusion. Owing to its accessibility and ease of use, ECO_2_Rs are typically performed in conventional H‐cell devices. In this double‐cell configuration, the anode and cathode are separated by a proton‐exchange membrane (such as a Nafion membrane) to prevent the anode from being poisoned by the products formed on the cathode and to prevent the products on the anode from being reverse oxidized.^[^ ^148^ ^]^ In an H‐type electrolyzer the ECO_2_R takes place on the cathode submerged in CO_2_‐saturated electrolyte and the products can be identified using a variety of analytical techniques, such as nuclear magnetic resonance (NMR) spectrometry, gas chromatography (GC), or liquid chromatography–mass spectrometry (LC‐MS). However, the overall process is severely restricted in terms of gas mass transport, which results in low current density (often less than 100 mA cm^−2^) and prevents further practical deployment.^[^ ^149^ ^]^

**Figure 9:**
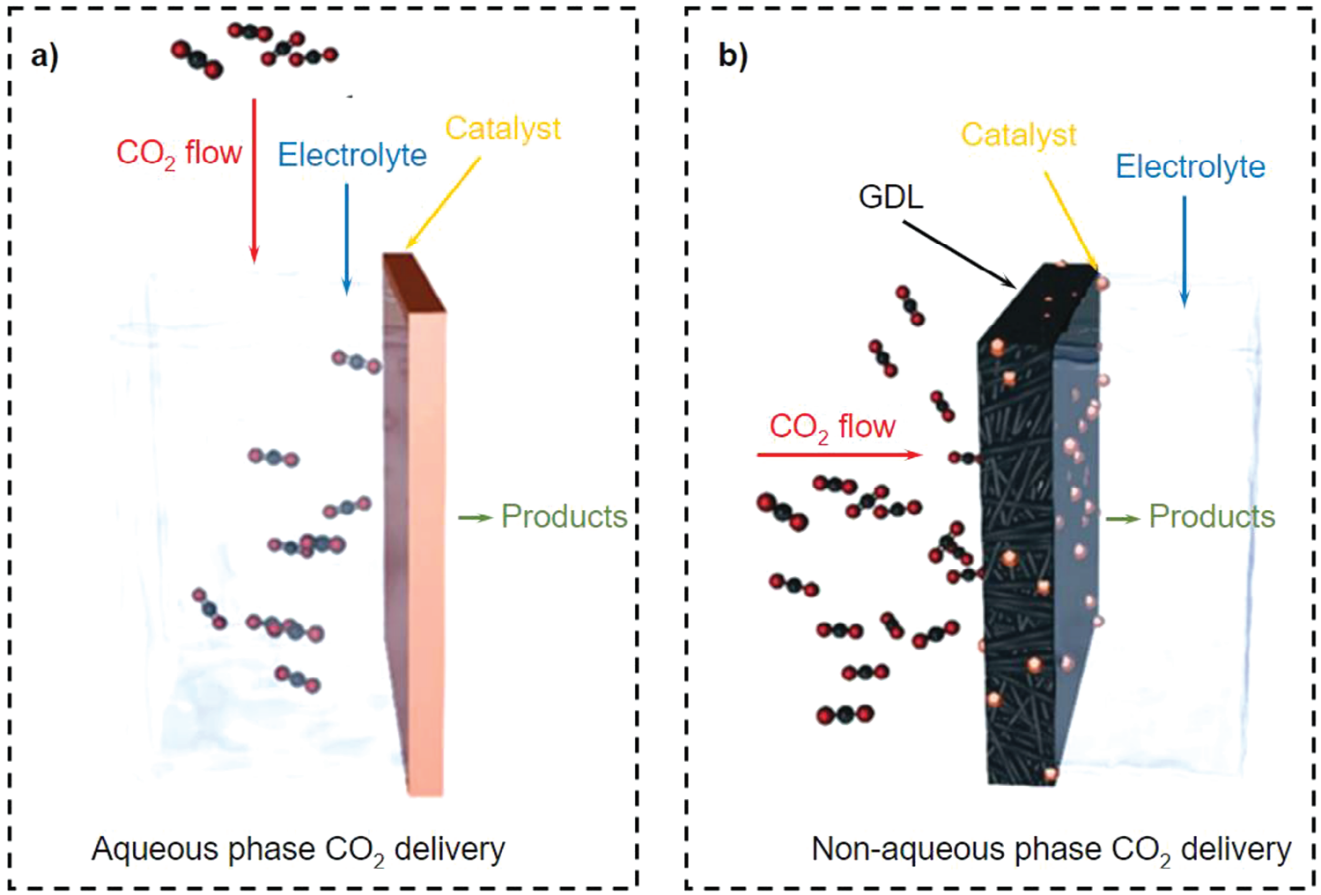
Illustration of ways to deliver CO_2_ from aqueous phases, and via GDLs in non‐aqueous phases. Adapted with permission.^[^ ^152^ ^]^ Copyright 2019, American Chemical Society.

Flow reactors are used for the ECO_2_R to increase the electrolysis current density.^[^ ^22^, ^150^ ^]^ To enhance ECO_2_R, gas diffusion electrodes (GDEs) have been developed as electrochemical reactors. The aim is to achieve high efficiency and good durability at high current densities (4200 mA cm^−2^), which is not attainable in conventional H‐cells (**Figure** **9**a).^[^ ^149^, ^150^ ^]^ Parallel to changing the hydrophobicity of the catalyst to influence product distribution (Figure 8), the use of a hydrophobic gas diffusion layer (GDL) as part of the GDE can contribute to tuning and optimizing these systems. Hydrophobic GDLs may swiftly supply CO_2_ to the catalyst surface without having to diffuse a great distance through the electrolyte (Figure 9b).^[^ ^149^, ^151^, ^152^ ^]^

It will not be immediately possible to use electrocatalysts flow‐cell CO_2_ electrolyzers on a large scale until significantly more research is performed; in particular, the issue of long‐term stability with high efficiency needs to be addressed to meet the performance standards for commercialization. The construction of the GDE, the membrane, and the flow field must all be optimized for the flow cell to avoid problems like water flooding, catalyst exfoliation, and the evolution of the salt build‐up on the GDE after prolonged electrolysis.^[^ ^153^ ^]^

In addition to these experimental design parameters to be considered, ECO_2_R is an extremely intricate reaction with several further factors to consider for optimization.

#### 3.2.4 Potential Used

Thermodynamic equilibrium potentials for CO_2_ reduction toward diverse products are close to 0 V versus RHE (Table 4). The high stability of CO_2_ (requiring large activation energies to convert) and slow kinetics of the reaction, however, necessitate the application of larger negative potentials to start the ECO_2_R and attain high current density. An excessive overpotential means extra energy input above the energy specified by thermodynamics, which leads to lower energy efficiencies.^[^ ^154^, ^155^ ^]^

#### 3.2.5 Hydrogen Evolution Reaction (HER) Side Reaction

Protons are required for the processes of reduction and hydrogenation in the ECO_2_R. However, protons can also be easily reduced to produce hydrogen, a process known as the HER.^[^ ^156^ ^]^ Since the ECO_2_R and HER have similar equilibrium potentials (Table 4), the HER competes with the ECO_2_R, decreasing the ECO_2_R's current efficiency.^[^ ^155^, ^157^ ^]^ The HER will be favored over the ECO_2_R if a catalytic site binds *H more strongly than *C (or *O), suppressing the adsorption of *COOH (or *OCHO), and promoting the adsorption of *H. A suitable ECO_2_R catalyst should, therefore, bind weakly with *H and bind strongly with *C (or *O).

#### 3.2.6 Binding Strength

A perfect catalyst should provide a reaction pathway that requires little to no energy to initiate.^[^ ^158^, ^159^ ^]^ A good catalyst should, in accordance with the Sabatier principle, have an optimal binding strength between the major reaction intermediates and the catalyst's surface sites. If the binding is too weak, the intermediates may not be able to bind to the catalyst's surface, and the reaction will not occur. On the other hand, if the binding strength is too high, the intermediates may become trapped on the catalyst's surface, leading to catalyst poisoning and the loss of its catalytic activity.

### 3.3 CMPs for ECO_2_R

The use of CMPs in ECO_2_R presents a new area of research; consequently, there are not many published studies where CMPs are used as electrocatalysts. Currently, metal‐based and hybrid CMP systems containing conductive carbon materials are the most common types of reported CMPs for the ECO_2_R, as discussed below.

#### 3.3.1 CO Production

The pathway to produce CO by ECO_2_R is less complex than that for the formation of other products, since it only requires a two‐electron transfer process to take place (see Table 4). ECO_2_R to CO is attractive for industrial purposes, as CO is a chemical feedstock that is widely employed in industry; it is a crucial component of syngas and a source of methanol and ammonia production.^[^ ^160^ ^]^ It is noteworthy that for CMP systems covered in this review, the main product of CO_2_ reduction is CO, owing to the relatively simple 2e^−^ transfer process.

**Scheme 6:**
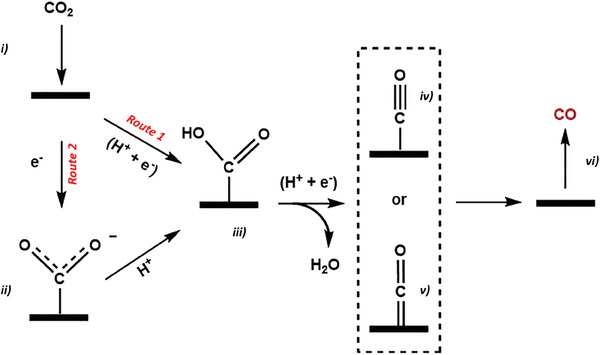
Potential mechanisms for the conversion of CO_2_ to CO. Adapted with permission.^[^ ^125^ ^]^ Copyright 2017, Elsevier.

Based on experimental observations and density functional theory (DFT) calculations, two potential reaction routes for the generation of CO have been suggested in both metal‐containing and metal‐free systems (**Scheme** **6**). Route 1 proposes the concurrent proton–electron (H^+^/e^−^) transfer from the electrolyte to the adsorbed CO_2_ resulting in the formation of a carboxyl intermediate (*COOH, Scheme 6, *iii*); then, a second H^+^/e^−^ attacks the oxygen atom of OH in the *COOH, producing H_2_O and a *CO intermediate (Scheme 6‐*iv* or *v*); the final step is desorption of *CO (Scheme 6‐*vi*) from the electrode.^[^ ^161^ ^]^ The conversion of *COOH into *CO during this process is easily accomplished. However, the first step of creating the *COOH is constrained by weak COOH binding, and the last step of *CO desorption is hindered by strong CO binding. Thus, the rate‐determining steps are these two processes that are affected by binding energy.

Route 2, the alternative pathway, proposes a decoupled electron and proton transfer process to produce *COOH, in which the adsorbed CO_2_ first accepts an electron to produce a CO_2_^−.^ radical (Scheme 6‐*ii*) and then a proton to produce *COOH; the formation of the CO_2_^−.^ radical is the rate‐determining step for this proposed route.^[^ ^161^, ^162^ ^]^ According to theoretical studies, a catalyst that is effective at reducing CO_2_ to CO should be able to both stabilize COOH* and destabilize CO*.

Post‐synthesis metalation is a frequently used approach to produce metallized CMPs. These CMPs typically include functional groups, like bipyridine units, that can interact with metals and enable the modification with metal ions. The integration of the catalytic center into the rigid CMP framework prevents catalyst dimerization; prior investigations^[^ ^163^, ^164^ ^]^ revealed that dimerization could still take place when flexible linkers were used or catalyst aggregates were formed. For example, to create an efficient electrocatalyst for ECO_2_R of CO_2_ to CO, Smith et al.^[^ ^165^ ^]^ inserted a *fac*‐[Mn(bpy)(CO)_3_^−^ Br] moiety into a CMP framework. SH cross‐coupling was used to synthesize the amorphous polymer CMP‐(bpy)_20_, which was then refluxed with [Mn(CO)_5_Br] to form CMP‐(bpy)_20_‐Mn with a surface area of 549 m^2^ g^−1^ (**Table** **5**, Entry 3). CO_2_ uptake was investigated since efficient CO_2_ adsorption is considered as a precondition for highly effective ECO_2_Rs. CMP‐(bpy)_20_‐Mn exhibits moderate CO_2_ adsorption (4.7 wt.%) at 298K, 1 bar. Under the same conditions, BPL carbon, a typical reference material, takes up 8.4 wt.% of CO_2_.^[^ ^166^ ^]^

**Table 5:** Summary of CMP‐based electrocatalysts for ECO_2_R.

Entry	Polymeric material	BET [m^2^g^−1^]	Conductive additive	Electrolyte	Applied potential [V]	Main product [FE %]	References
1	PF‐5 film	1000	–	0.1 m Et_4_NBF_4_/acetonitrile solution	n.r.	CO	[167]
2	PyPOP@G	582.7	Graphene	0.1 m KHCO_3_	−1.0 (vs. RHE)	n.r.	[168]
3	CMP‐(bpy)_20_‐Mn	549	–	0.06 m Phosphate buffer	−1.6 (vs. Ag/Ag^+^)	CO (0.43%)	[165]
4	CNS‐NiSA	458	CNSs	0.5 m KHCO_3_	−0.8 (vs. RHE)	CO (95%)	[169]
5	CNT@CMP (CoPc‐H_2_Pc)	70	CNTs	0.5 m KHCO_3_	−0.9 (vs. RHE)	CO (97%)	[170]
6	COP‐SA	106.33	carbon black	0.5 m KHCO_3_	−0.65 (vs. RHE)	CO (96.5%)	[171]
7	Pt/TPE‐CMP	360	CNTs	0.5 m KHCO_3_	−1.5 (vs. Ag/Ag+)	(C1‐C8) (>95%)	[172]
8	CoPPc‐TFPPy‐CP	464.9	–	0.1 m KCl	−1 (vs. RHE) −1.2 (vs. RHE)	C_2_H_5_OH 43.25% HCOOH 13.91%	[16]
9	BNPI‐1 (NaF 0.99) BNPI‐1 (NaI 0.66) BNPI‐2	54 728 26	–	0.1 m KHCO_3_	0.03 (vs. RHE) −0.26 (vs. RHE) −0.26 (vs. RHE)	HCOOH 91% CH_3_OH 85% HCOOH 45% CH_3_OH 67%	[17]
10	pPI‐1 pPI‐2	20 342	–	0.1 m KHCO_3_	‐0.76 (vs. RHE) −0.26 (vs. RHE) −0.56 (vs. RHE) −0.26 (vs. RHE)	HCOOH 14% CH_3_OH 52% HCOOH 20% CH_3_OH 95%	[73]

Et_4_NBF_4_ ‐ Tetraethylammonium tetrafluoroborate.

The main goal of the work by Smith et al. was to show the feasibility of the strategy by demonstrating that the Mn center still possesses electrochemical activity within the CMP framework. The electrode was not optimized in any way to facilitate catalysis, which is the reason for the extremely low FEs for CO production (0.43%) and the predominance of the HER. To tackle these issues the authors were aiming to use the route suggested by Kubiak and co‐workers,^[^ ^173^ ^]^ where they demonstrated that Mg^2+^ can significantly speed up (by over tenfold) the pathway in solution, enabling activity at lower overpotentials (0.3–0.45 V). Moreover, to improve catalytic performance of the CMP‐(bpy)_20_‐Mn, the authors suggested the use of GDEs.

Since CMPs often have weak electronic conductivity, graphene or carbon nanotubes are typically used with CMPs to improve their capacity to transfer electrons. For instance, by using the SH cross‐coupling reaction, Soliman et al.^[^ ^168^ ^]^ designed the microporous composite material PyPOP@G (Table 5, Entry 2), which exhibits considerable electrocatalytic activity for ECO_2_R (5 mA cm^−2^ at −1.0 V vs RHE). Owing to the sufficient basic sites of the included pyrimidine rings, which have a favorable binding energy toward CO_2_, PyPOP served as the catalytic center for ECO_2_R, while graphene provided enhanced conductivity (although no values are provided for the reported catalyst). Further details of products formed during the reported catalytic process were not reported on, as only preliminary electrochemical investigations were carried out.

Another example of a synergistic CMP and supporting material combination as ECO_2_R catalyst was reported by Zhao et al.^[^ ^169^ ^]^ in 2021. A novel sandwich‐like CMP was synthesized utilizing an ultrathin exfoliated nickel phosphorus trisulfide (NiPS_3_) 2D template (to generate N and S co‐coordinated Ni sites) with the CMP formed via polymerization directly onto the template. These porous polymer nanosheets were pyrolyzed and then decomposed to form a porous carbon nanosheet (CNS)‐based catalyst with S and P dopants and single distributed Ni atoms (CNS‐NiSA). CNSs possess higher aspect ratios, higher surface areas and longer‐distance conductivity than regular porous carbons, effectively exposing single‐atom sites and shortening the distance over which the electrolyte diffuses, thus boosting electrocatalyst performance. The resultant porous CNSs' show exceptional efficacy as electrocatalysts for ECO_2_R (95% FE for reduction of CO_2_ to CO, Table 5, Entry 4). To explore this reaction further, a typical three‐electrode H‐type cell was used to investigate the ability of the CNS‐NiSA to electrochemically convert CO_2_. The activity of CNS‐NiSA had a greater total current density (j) in the CO_2_‐saturated electrolyte (as opposed to an Ar‐saturated electrolyte). CNS‐NiSA demonstrated a high FE_CO_ of 90%, with the maximum FE_CO_ of almost 95% at −0.80 V versus RHE over a wide potential range. Furthermore, the CO_2_‐to‐CO TOF of 1824 h^−1^ at −1.0 V versus RHE was equivalent to that of reported Ni‐based catalysts.^[^ ^174^, ^175^ ^]^ Since the production of the COOH* intermediate has a high free energy barrier, the authors hypothesized that the first electron transfer (from CO_2_ to COOH*) was the rate‐limiting step. Additionally, FE_CO_ and j_CO_ showed only little sign of degradation during the stability test performed at −0.8 V versus RHE, demonstrating that CNS‐NiSA remains catalytically active.

To ascertain whether the high ECO_2_R activity of CNS‐NiSA was caused by the Ni‐N_3_S single‐atom sites, the N─C (Ni‐free catalyst) was prepared and evaluated alongside a SCN^−^ poisoning experiment (the SCN‐ interacts and blocks the Ni active sites). The FE_CO_ and j_CO_ values for N─C were significantly lower after poisoning than those of CNS‐NiSA, indicating that the Ni‐N_3_S single‐atom moieties are the real active sites and are essential for effective ECO_2_R.

Wang et al.^[^ ^170^ ^]^ designed and prepared an ultrathin CMP sheath around CNTs using a solid‐state ionothermal copolymerization of CoPc and metal‐free H_2_Pc using a Scholl reaction. The excellent electrical conductivity of carbon nanotubes (CNTs) has made them a popular choice for catalytic supports that encourage electron transport. The composite exhibits high FEs (up to 97%) for the conversion of CO_2_ to CO across a wide range of potentials (−0.5 to −1.0 V vs RHE), an excellent TOF (97 592 h^−1^ at −0.65 V) and a high current density (> 200 mA cm^−2^) owing to the synergy of H_2_Pc moieties serving as proton/electron dual donors.

**Figure 10:**
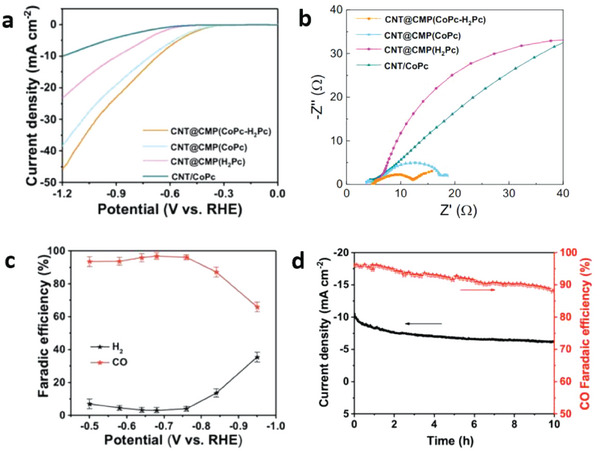
Polarization curves a) and Nyquist plots b) of CNT@CMP(CoPc‐H_2_Pc), CNT@CMP(CoPc), CNT@CMP(H_2_Pc), and CNT/CoPc. FEs for the CNT@CMP(CoPc‐H_2_Pc) in a flow cell c) and current density and FE_CO_ of CNT@CMP(CoPc‐H_2_Pc) for 10 h d). Adapted with permission.^[^ ^170^ ^]^ Copyright 2021, Wiley.

A standard two‐compartment H‐cell electrochemical setup was used to test the ECO_2_R ability of CNT@CMP(CoPc‐H_2_Pc), where CoPc and H_2_Pc represent porphyrin additives with or without cobalt, respectively. Control materials, namely CNT@CMP(CoPc), CNT@CMP(H_2_Pc) and CNT/CoPc, were synthesized for comparison. CNT@CMP(CoPc‐H_2_Pc) showed the maximum current density of ‐45 mA cm^−2^ (**Figure** **10**a). The Nyquist plots, which provide insight into the stability of the catalysts, also demonstrated that CNT@CMP(CoPc‐H_2_Pc) had the highest conductivity and electroactivity (Figure 10b). As the current density of ECO_2_R in three‐electrode H‐type cells is restricted by low CO_2_ solubility and slow diffusion, the authors used flow cells and achieved high current densities for CNT@CMP(CoPc‐H_2_Pc). The CNT@CMP(CoPc‐H_2_Pc) had a high FE_CO_ of 96% at a low overpotential of −0.65 V (Figure 10c) with a maximum TOF_co_ of 97 592 h^−1^, indicating that more active sites are participating in the ECO_2_R than HER using CNT@CMP(CoPc‐H_2_Pc). The CNT@CMP(CoPc‐H_2_Pc) catalyst had high FEs in a broad potential range between −0.5 and −1.0 V (vs RHE) and maintained its function during at least 10 h of electrolysis; the FEs and the current density decreased slightly over 10 h (Figure 10d).

The combination of CoPc and H_2_Pc is essential for enhancing the catalysts' activity and stability. The overpotential of CoPc/H_2_Pc is substantially lower than that of CoPc/CoPc. The added H_2_Pc prevents the CoPc moiety from demetallizing and boosts the proportion of atomically dispersed Co sites in the CMP. Also, this difference is linked to an increase in the nucleophilicity of Co sites imposed by the H_2_Pc moiety. Moreover, the H_2_Pc unit functions as a proton donor by transferring hydrogen to the Co active sites in CNT@CMP(CoPc‐H_2_Pc) via the pyrrolyl moieties, stabilizing the reactive CO_2_ intermediates by H‐bonding interaction. Overall, activity for these materials is similar to other reported electrocatalysts based on CoPc,^[^ ^176^, ^177^, ^178^, ^179^ ^]^ and even to metals like Au and Ag.

In 2021, Song et al.^[^ ^171^ ^]^ showcased another noteworthy demonstration of employing a cobalt‐based electrocatalyst for the reduction of CO_2_ into CO. They presented a CMP‐based ultrathin material composed of a cobalt‐containing tetraamino phthalocyanine‐squaraine‐based CO_2_ reduction catalyst, abbreviated as COP‐SA (Table 5, Entry 6). This material exhibited remarkable performance characteristics, including a high current density of 9.74 mA cm⁻^2^ with 96.5% selectivity for CO production. Moreover, it demonstrated a TOF of 165 600 h^−1^ at −0.65 V versus RHE. The unique ultrathin structure of COP‐SA was pivotal in enhancing its catalytic properties. This design increased the accessibility of active cobalt sites, while the extended π‐conjugated structure facilitated efficient electron transfer during the ECO_2_R reaction. Theoretical investigations further elucidated the mechanism behind the improved performance. The presence of squaraine units in COP‐SA weakened the binding strength of CO to the cobalt atoms, expediting the desorption of reaction products. This effect played a crucial role in enhancing the overall catalytic efficiency of the material.

Electropolymerization (EP) of electrocatalysts enables thin films to develop directly on the electrode and minimizing the distance between the films and the electrode, improving the ability of CO_2_ to achieve close contact with active electrodes. The porous nature of the EP CMP sheets makes them attractive electrocatalysts with enhanced reactant diffusion and electron transport capabilities. Hu et al.^[^ ^167^ ^]^ reported the use electropolymerization to prepare microporous carbazole‐functionalized films of iron porphyrins for ECO_2_R for CO production (Table 5, Entry 1). In addition to close electrode contact, the porous design of the film also offers opportunities for dispersion of the supporting electrolyte and solvent, as well as for efficient charge transport. Disappointingly, a 30% reduction of the catalyst's activity after only four voltage sweeps during the ECO_2_R process indicated that long‐term usage is still a significant challenge for this system. The authors propose that this decline is due to the carbon atoms of the porphyrin ring being partially carboxylated, and aim avoid this issue in the future by substituting the peripheral phenyl groups with bulkier groups. In their early study, Saveant and co‐workers^[^ ^180^ ^]^ found that iron porphyrins were unstable during homogeneous ECO_2_Rs. Despite the deactivation of the catalyst, recent studies^[^ ^181^, ^182^ ^]^ demonstrate that immobilization of iron‐porphyrin films can be a successful approach for ECO_2_Rs if substantial surface coverage of the iron porphyrin (≈10^−8^ mol cm^−2^) can be obtained.

As already discussed, the effectiveness of some CMPs for CO_2_ reduction was enhanced by adding transition metals. This approach was also investigated by Strasser et al.^[^ ^183^ ^]^ in 2017. They studied a series of porous carbons that were transition‐metal‐ and N co‐doped. These materials contained atomically dispersed M‐Nx centers (M = Mn, Fe, Co, Ni, Cu), serving as the active sites for the efficient ECO_2_R to CO. They investigated the catalytic activity of these materials and found that the ECO_2_R performance of M─N─C (metal─nitrogen─carbon) electrocatalysts, particularly Ni─N─C, was comparable to that of Au‐ and Ag‐based electrocatalysts. M─N─C electrocatalysts contain transition metals that are atomically distributed (M = Fe, Co, Ni, etc.) and a nitrogen‐doped carbon matrix. Owing to their maximum atomic utilization, variable coordination environments and distinctive electronic features, single‐atom carbon‐based catalysts have demonstrated exceptional performance for CO_2_‐to‐CO conversion.^[^ ^184^ ^]^

#### 3.3.2 Non CO Products

Although the routes to ECO_2_R to CO represent advances in CO_2_ utilization, it would be beneficial to create more reduced species, especially those with longer carbon chains (Table 4). To achieve this goal, it is essential to create CMPs that have active sites with adequate adsorption energies for chemical intermediates to selectively form diverse products via various CO_2_ reduction routes (Scheme 5 and Table 4). Some CMPs that are able to achieve this goal are discussed below.

Ampelli et al.^[^ ^172^ ^]^ synthesized tetrakis‐phenylethene (TPE) CMPs using a Yamamoto homo‐coupling reaction, and modified these materials by a so‐called sol immobilization treatment to add noble (Pt) and non‐noble (Fe) metal nanoparticles to form the active catalytic sites. Their effectiveness was evaluated using a custom‐built laboratory scale electrocatalytic system that utilized a gas diffusion membrane (GDM). Compared with the more in‐depth researched liquid‐phase electrochemical systems discussed earlier (see H‐cell set up in Figure 9a), the designed electrochemical cell offered several advantages, including eliminating solubility issues of CO_2_ and the requirement to recover liquid products, thus increasing productivity and selectivity for more complex products. It is noteworthy that long‐chain hydrocarbons (C1‐C8) were found in all investigations, along with the production of hydrogen, carbon monoxide, and methane (Table 5, Entry 7); all experiments had very high FEs (>95%). The total productivity of the Pt/TPE‐CMP electrocatalyst, which is defined as the sum of the liquid products, was increased with the addition of CNTs (30 wt.%). The results for the non‐noble Fe/TPE‐CMP electrocatalyst were less encouraging, attributed to the relatively large size of the Fe nanoparticles. Moreover, the authors stressed the need to localize the Pt active phase on the polymer surface where CO_2_ adsorption takes place, implying that the rate‐determining step could be the creation of the anion radical CO_2_^·−^. This study provides opportunities for the design of hybrid, metal‐containing materials for future catalytic uses.

Cobalt phthalocyanine (CoPPc) represents another promising material for catalyzing the electrochemical reduction of CO_2_ owing to its high product selectivity and easy availability. Jiang et al.^[^ ^16^ ^]^ have designed and synthesized a novel CoPPc‐based pyrene‐containing porous conjugated polymer CoPPc‐TFPPy‐CP. Pyrene, a widely investigated building block, contains a highly symmetrical four‐benzene‐ringed polycyclic aromatic structure that offers unique optoelectronic properties.

The BET surface area of CoPPc‐TFPPy‐CP was 464.9 m^2^ g^−1^, with a type IV isotherm indicating mesoporous character, which facilitates mass transport during the generation of the CO_2_‐to‐liquid products. Tuning the cobalt to a higher oxidation state enhances the catalytic performance in CO_2_ reduction. The elevated Co oxidation state affects the spin state of the Co center, which in turn tunes the electron distribution and orientation of Co‐3d orbitals. These changes resulted in increased CO_2_ adsorption and improved catalytic activity. The cobalt center with a higher oxidation state also shows a larger tendency to attract and activate the Lewis basic O‐site in the generated carboxyl group, which is favorable for C─C dimerization. The mesoporous nature of the polymer and the presence of highly efficient active sites produce an efficient catalyst for the electrochemical reduction of CO_2_. A FE of 43.25% at −1.0 V versus RHE was achieved for the formation of ethanol, and a formic acid FE of 13.91% at −1.2 V versus RHE using CoPPc‐TFPPy‐CP. Moreover, this porous polymer is the first reported organic polymer that shows selectivity to liquid products from CO_2_ reduction.

In all of these discussed systems, the presence of metals was required to facilitate or act as catalysts. Only a small number of studies have been published where no metals or co‐catalyst were required or present in the conversion of CO_2_.

Our group recently synthesized a series of porous polyimides (pPIs) using a metal‐free condensation reaction while applying the Bristol–Xi'an‐Jiaotong (BXJ) approach, which allows for the optimization of surface areas, pore sizes and related physical properties.^[^ ^17^ ^]^ The structures of the resultant pPIs, based on 1,4,5,8,‐naphthalenetetracarboxylic dianhydride and melamine in the case of BNPI‐1, and tris‐(4‐aminophenyl)triazine in the case of BNPI‐2, respectively, are shown in Figure 6. These were utilized as electrocatalysts, free from co‐catalyst‐ and metals, for the conversion of CO_2_. By selectively tuning the porosity properties and surface areas of the pPIs, we were able to selectively reduce CO_2_ to either formate or methanol with excellent FEs of 91% and 85%, respectively. The research revealed that the surface area of the material strongly influences the pathway of CO_2_ reduction to product formation, with higher surface areas demonstrating superior electrocatalytic performance for methanol production. Moreover, the presence of larger pores in the mesoporous region resulted in an increased propensity to form methanol.

In further studies, the versatility of two previously mentioned co‐catalyst‐ and metal‐free pPI systems designed by our group (named pPI‐1 and pPI‐2, see Section 2.2.3, Figure 6 for structures), was recently explored for the conversion of CO_2_.^[^ ^73^ ^]^ The results show FEs of 95% for methanol and 20% for formate production, respectively, for these perylene‐based systems. The higher efficiency for methanol formation could be attributed to the higher surface area and broad pore size distribution of the pPIs.

This exciting approach offers promising opportunities to produce useful fuels and feedstocks from CO_2_ – in a metal‐ and co‐catalyst‐free reaction environment. Furthermore, careful and selective tuning of the products by only changing physical properties of the porous polymer offers exciting opportunities for the exclusive use of organic porous catalyst to produce higher carbon products, thus addressing global challenges in a long‐term sustainable fashion. It is important to consider the cost effectiveness of these materials. Moves in our group toward materials that are metal‐free, require little purification and are chemically and thermally stable (therefore making them reusable) represent significant progress in this field. It is important to note that the synthesis of the materials is metal‐free as well, thereby stopping the formation of metal‐containing liquid waste that requires further treatment. A back‐of‐the‐envelope estimation of the cost of our materials (based on a research‐scale synthesis, no scale‐up or process engineering input, and starting materials bought from regular laboratory chemical suppliers, not bulk chemical prices) lies between $30 and $35 per gram; it is noteworthy that these costs are for metal‐free catalysts that can be used multiple times.

## 4 Conclusion

An urgent need for action from the scientific community exists to provide solutions for the global grand challenges faced by humanity. In this review we have shown that CMPs exhibit significant potential to address the challenge of increasing CO_2_ concentrations through CO_2_ capture and conversion to valuable fuels and feedstocks. Urgent development of this class of materials will add to the wide range of technical solutions that are under development to create a truly circular carbon economy.

However, despite substantial advances in recent years, the field of CO_2_ conversion using CMPs is still in its nascent stage of development. Therefore, in the realm of practical applications, several critical challenges remain to be addressed in the field.

Currently, there are no standardized and normative criteria for catalytic measurements, making it difficult to objectively evaluate the efficacy of different CMPs and determine routes toward superior materials. Providing comprehensive test data, including catalyst loading, stability, and turnover frequencies would enable thorough comparisons, evaluation, and optimization of designs. The development of standardized test methods and establishing a unified evaluation system for future research studies to overcome these challenges and promote consistent and reliable assessment of CMP catalytic performances is therefore urgently required. In addition, selectivity of CMPs toward CO_2_ over other gases, during both the capture and conversion stages, is not fully explored in the literature. The ability of these materials to operate as catalysts in both atmospheric and (simulated) industrial environments needs to be understood and explored as part of such standardized test regimes.

The cost effectiveness of these CMPs as CO_2_ reduction catalysts has not been addressed in the literature; such technoeconomic analyses will need to be performed if commercially viable systems are to be created.^[^ ^185^, ^186^ ^]^ When considering economic factors for scaling up for industrial applications, careful monitoring of the long‐term durability of heterogeneous catalysts by repeat tests of catalytic performance need to be standardized.

Detailed understanding of the catalytic mechanism involved in CO_2_ transformation, especially for multifunctional co‐catalyst‐ and metal‐free organic catalysts, will be crucial for the development of highly efficient and selective catalysts. Such mechanistic investigations should be undertaken in close collaboration with computational scientist, also employing the latest available tools for a digital approach to catalyst discovery and mechanism elucidation.

In addition to these recommendations, the following suggestions for future investigations should be considered.

Although H‐type cells in aqueous media are commonly employed to study catalytic mechanisms, the current density is often constrained due to the limited solubility of CO_2_. GDE‐assisted flow cells and fuel cells have shown promise in efficiently delivering CO_2_ to the electrode surface, enabling industrial‐level reduction of CO_2_. However, challenges such as flooding and clogging of the gas diffusion layer (GDL) need to be addressed before long‐term and practical applications will be achieved. In the move toward future direct reduction of CO_2_, commercial‐scale applications will crucially require development and optimization of reactor design, especially for GDEs.

In general, the advancement of dependable in situ and online characterization techniques holds great promise and is highly desirable in tackling critical challenges in the field of ECO_2_RR catalysis.^[^ ^187^, ^188^, ^189^ ^]^ Use of different in situ methods during electrocatalytic reactions, including surface‐enhanced Raman spectroscopy, surface‐enhanced infrared absorption spectroscopy, and X‐ray absorption spectroscopy, present opportunities to elucidate the intricate processes and complex reaction mechanisms. Combining these techniques can enable more precise monitoring of the electrocatalytic interface and offer more compelling evidence to validate hypotheses. Such operando techniques for studying the ECO_2_RR can eliminate the laborious steps involved in conventional methods for analyzing complex multi‐product mixtures, and can provide an efficient way to screen for ECO_2_RR catalysts with desired properties.

Finally, significant opportunities exist to explore the coupling of further precursors and reagents for reaction with reactive species generated during CO_2_ reduction, and to exploit valuable oxidation reactions at the anode.^[^ ^190^ ^]^ It is important to note that any electricity used in ECO_2_RR in scale up versions of these processes in the future must come from true green sources (rather than those that generate more CO_2_) for this process to be viable.

While there is still much to be accomplished in the realms of electrochemical and chemical catalysis, we anticipate that the comprehensive overview of CMP catalysts presented in this work will contribute to a deeper understanding of this emerging class of functional materials. Furthermore, the hope is that this topical overview of an area of global importance will provide guidance to address these challenges, contribute to shaping future research endeavors, and enable exploration of new routes to CO_2_ utilization.

## Conflict of Interest

The authors declare no conflict of interest.
